# Sensing of extracellular l-proline availability by the integrated stress response determines the outcome of cell competition

**DOI:** 10.1126/sciadv.adw1883

**Published:** 2025-07-09

**Authors:** Shruthi Krishnan, Ana Lima, Sizhe Tan, Ying Thong Low, Salvador Perez Montero, Aida Di Gregorio, Adrian Perez Barreto, Sarah Bowling, Karen Vousden, Tristan A. Rodriguez

**Affiliations:** ^1^ National Heart and Lung Institute, Imperial College London, London, UK.; ^2^ The Francis Crick Institute, London, UK.; ^3^ Department of Developmental Biology, Stanford University School of Medicine, Stanford, CA, USA.

## Abstract

Cell competition is a conserved fitness quality control that eliminates cells that are less fit than their neighbors. How winner cells induce the elimination of losers is poorly understood. We tackle this question by studying the onset of embryonic differentiation in mice, where cell competition eliminates 35% of embryonic cells. These loser cells have mitochondrial dysfunction, which we show causes amino acid deprivation and activation of the integrated stress response (ISR), a pathway essential for their survival. We demonstrate that l-proline is a key amino acid sensed by the ISR and that proline represses the ISR and drives their elimination. These results indicate that cell competition acts as a previously unidentified tissue-sparing mechanism, regulated by the availability of extracellular amino acids, that allows for the elimination of dysfunctional cells when amino acids are plentiful but ensures their survival in nutrient-poor environments.

## Teaser

Cell competition acts as a tissue-sparing mechanism, eliminating dysfunctional cells when amino acids are plentiful.

## INTRODUCTION

There is an increasing awareness that how cells respond to their environment is shaped not only by their intrinsic genetic composition but also by the nature of the cells that surround them. One example of this is cell competition, a fitness quality control that results in the elimination of cells that are potentially viable (losers) but that are less fit than their neighbors (winners) (*1*). Cell competition has been described from *Drosophila* to humans and shown to modulate the overall fitness of a tissue in a broad set of contexts (*2*–*6*). These include, for example, the elimination of aberrant cells during embryonic development or aging or the expansion of cancerous cells. Cell competition does not always target defective cells, but in a variation termed supercompetition, some cells can gain a selective advantage by actively eliminating wild-type (WT) cells. For example, cells with MYC overexpression (*7*–*9*), loss of function of p53 (*10*–*12*), or karyotypic abnormalities (*13*) have all been shown to eliminate their WT neighbors through competition (*14*). A key question is how cells sense their relative fitness levels. However, the mechanisms by which cells with different fitness levels recognize each other are still poorly understood.

To start addressing this problem, we studied the competition that occurs at the onset of pluripotent stem cell differentiation. We have shown that during early mouse postimplantation development, about 35% of cells are eliminated through cell competition, and the main features of this elimination can be captured in vitro during the differentiation of embryonic stem cells (ESCs) (*15*–*17*). Single-cell transcriptomic and functional analysis of the losers in the embryo identified that these cells showed mitochondrial dysfunction. Furthermore, we also demonstrated that manipulating mitochondrial activity is sufficient to change the competitive ability of pluripotent stem cells (*17*), pinpointing mitochondrial function as a key determinant of competitive cell fitness. However, how do cells respond to their relative mitochondrial activity?

Mitochondria play a broad range of roles in cellular function, from energy production, the generation of essential metabolites, such as amino acids or nucleotides, and determining the response to nutrient availability in the environment to the regulation of apoptosis or the redox balance of the cell (*18*, *19*). Consequently, mitochondrial dysfunction can lead to a variety of different stress responses. The analysis of the loser epiblast cells in the embryo showed that they presented a signature of activation of the integrated stress response (ISR). The ISR can be activated by diverse stress stimuli, including amino acid deprivation, proteostasis defects, viral infection, and redox imbalance. Once activated, the ISR reduces global translation via phosphorylation of eukaryotic translation initiation factor 2 subunit alpha (eIF2α) but allows selective translation of selected genes, most notably activated transcription factor 4 (ATF4), which acts to restore cell homeostasis (*20*, *21*). Here, we investigated the role of ISR/ATF4 in mouse cell competition and found that it is essential for the survival of cells with mitochondrial dysfunction. We show that in this context, the ISR is activated by amino acid starvation and promotes amino acid metabolism in defective cells. We find a key role for l-proline and identify that when cells with mitochondrial dysfunction are surrounded by WT cells, they are unable to sustain ISR activation due to the uptake of extracellular l-proline, which induces their elimination. These results indicate that in a heterogeneous cell population, WT and stressed cells respond to the microenvironment differently, and this difference affects tissue composition by determining whether stressed cells survive or die.

## RESULTS

### Loser cells show activation of the ISR

Here, we set out to determine how winner and loser cells communicate their fitness levels during cell competition occurring in the early mouse embryo, where we have previously shown it to be responsible for the elimination of about 35% of epiblast cells (*16*, *17*). Three possible modes of competition have been proposed—competition for nutrients, where loser cells are less able to take up essential nutrients, mechanical competition, where loser cells are eliminated at high densities due to their increased sensitivity to compaction, and fitness sensing, where winners and losers sense their relative fitness levels (*6*). To distinguish between these possibilities, we first studied the mode of elimination of mouse ESCs with defective bone morphogenetic protein (BMP) signaling (*Bmpr1a*^*−/*−^) cocultured with WT cells, as it recapitulates the most important features of the competition occurring in the embryo (*15*–*17*). WT and mutant cells were mixed in a 1:1 ratio and plated at increasing densities. Cells were then induced to differentiate for 4 days by culture in N2B27 (*15*). We found that although *Bmpr1a*^*−/*−^ ESCs were effectively eliminated at low densities, this elimination was inhibited as cell density increased (fig. S1). The decreased competitive elimination of defective cells at high densities suggests that neither mechanical force nor competition for nutrients is likely to determine the outcome of cell competition.

**Fig. 1.**
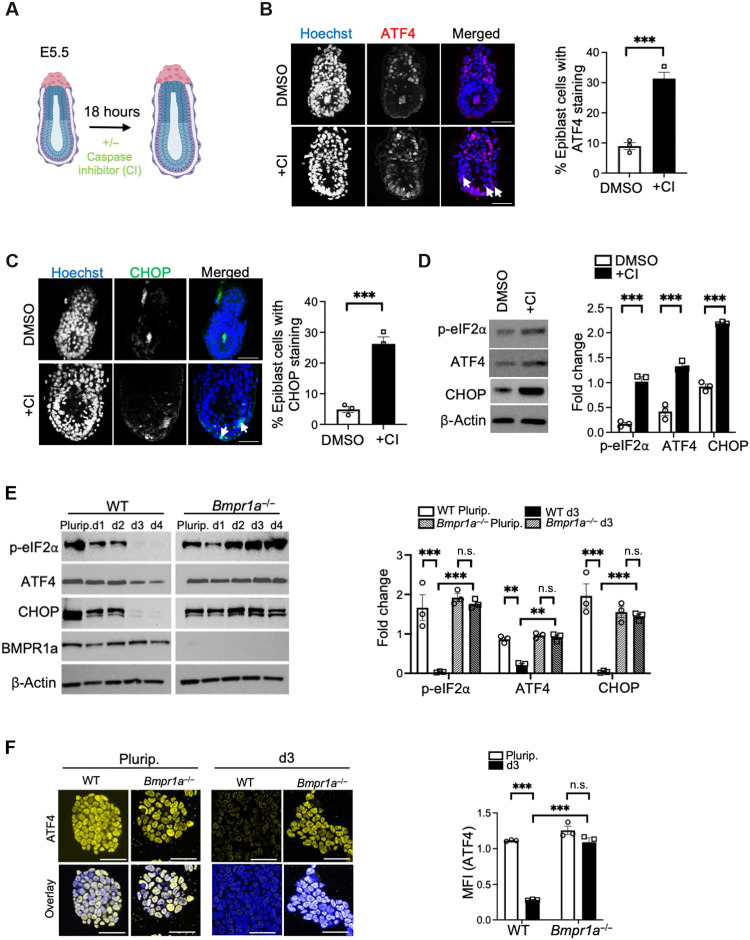
Defective cells eliminated by cell competition have a signature of ISR activation. (**A**) Experimental design. E5.5 embryos were treated with or without the pan-CI, Z-VAD(OMe)-FMK (Z-VAD-FMK) (100 μM), for 18 hours. (**B** and **C**) Immunofluorescence analysis of ATF4 [red; (B)] and CHOP [green; (C)] in E6.5 embryos treated with CI or DMSO vehicle control for 18 hours. Embryos were counterstained with Hoechst (blue). Scale bars, 50 μm. Bar graphs depict quantification of epiblast cells stained positive for ATF4 (B) and CHOP (C). (**D**) WT ESCs were treated at day 2 of differentiation with CI (100 μM) or DMSO for 24 hours, and immunoblots were performed to analyze the levels of ISR proteins: phosphorylated eIF2α (p-eIF2α), ATF4, and CHOP. β-Actin was used as a loading control. Bar graph represents densitometry-based quantification of fold change in the expression of each of the proteins as a value normalized to the respective loading control. (**E**) Immunoblot analysis of WT and *Bmpr1a^−/−^* cells after culture under pluripotency (Plurip.) conditions or at different time points of differentiation [day 1 (d1) to d4]. Expression of p-eIF2α, ATF4, CHOP, BMPR1a, and β-actin (loading control) is analyzed. Bar graphs represent fold change in expression levels of p-eIF2α, ATF4, and CHOP in ESCs and d3. n.s., not significant. (**F**) Immunofluorescence analysis of ATF4 (yellow) in WT and *Bmpr1a^−/−^* ESCs cultured under Plurip. conditions or at d3 of differentiation. Nuclei are counterstained with Hoechst (blue). Scale bars, 100 μm. Bar graph represents quantification of staining intensity of ATF4 [mean fluorescence intensity (MFI) in arbitrary units] in the cells. *n* = 3 for all studies. Error bars denote SEM. *P < 0.05, ***P < 0.005; unpaired *t* test [(B) to (D)] or two-way analysis of variance (ANOVA) and Tukey’s post hoc test [(E) and (F)].

Given the plausibility of the fitness sensing model, we hypothesized that one mechanism by which winner and loser cells could sense each other is through the differential activation of stress pathways. We have previously shown that a key feature of loser cells is their mitochondrial dysfunction (*17*). In the embryo, the cells eliminated by competition additionally display a transcriptional signature of ISR activation (*17*), a pathway downstream of several stresses associated with mitochondrial dysfunction, including amino acid deprivation, endoplasmic reticulum (ER) stress, and redox imbalance. To validate these results, we cultured embryonic day 5.5 (E5.5) embryos in the presence of a pan–caspase inhibitor (CI) or dimethyl sulfoxide (DMSO) vehicle control for 18 hours (Fig. 1A). After treatment, the embryos were stained for the ISR markers—ATF4 and C/EBP homologous protein (CHOP). We found both to be increased in the epiblast cells of CI-treated as compared to DMSO-treated control embryos (Fig. 1, B and C). This supports the idea that loser cells in the embryo exhibit ISR activation.

We next tested whether the ISR is activated in the cells dying during the first steps of ESC differentiation, as this models early postimplantation epiblast development (*15*). We differentiated WT ESCs for 3 days in N2B27, treated them with CI or DMSO for 24 hours, and assessed phosphorylated eIF2α (p-eIF2α), ATF4, and CHOP expression. We found increased levels of expression for all three ISR markers in CI-treated cells when compared to DMSO-treated controls (Fig. 1D), suggesting that the ISR is also marking the cells eliminated during differentiation. This led us to ask whether there is also a differential ISR response in the defective ESCs eliminated by cell competition. For this, we studied two loser cell models with mitochondrial dysfunction (*17*), the abovementioned *Bmpr1a^−/−^* ESCs and dynamin-related protein 1 (*Drp1^−/−^*) cells, that carry a null mutation in the regulator of mitochondrial fission, *Drp1*. In agreement with the high expression of p-eIF2α and ATF4 in the preimplantation embryo and ESCs (*22*), we found that WT and mutant cells display activation of ISR markers when cultured as homogeneous populations under pluripotency conditions (Fig. 1, E and F, and fig. S2, A and B). In contrast to this, from day 3 of differentiation, we found that while WT cells strongly down-regulate ISR protein expression, both defective cell types failed to do so and sustained robust activation of this pathway (Fig. 1, E and F, and fig. S2, A and B). The activation of the ISR in mutant cells is not due to these cells retaining pluripotency, as we have previously shown that at day 3 of differentiation, *Bmpr1a^−/−^* ESCs and *Drp1^−/−^* cells have down-regulated pluripotency factor expression in a similar way to controls (*15*, *23*). The fact that we only see competition from day 3 of differentiation (*15*–*17*) suggests that this differential ISR activation between winner and loser cells may regulate their competitive behavior.

### Activation of the ISR is essential for loser cell survival during cell competition

**Fig. 2.**
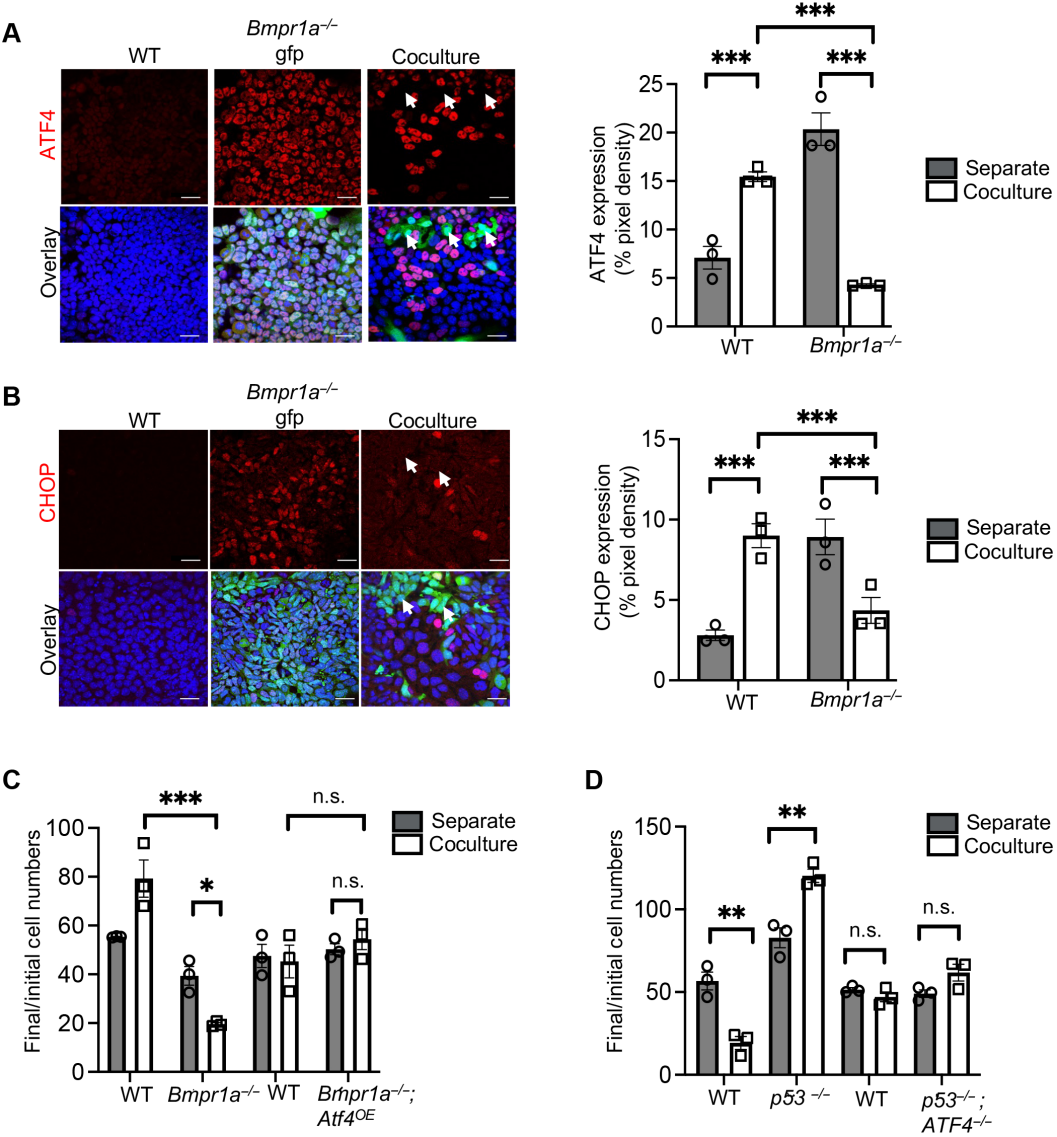
Activation of ISR is essential for loser cell elimination. (**A** and **B**) Immunofluorescence analysis of ATF4 and CHOP (red) in WT and *Bmpr1a^−/−^*–Gfp (green fluorescent protein) cells cultured under separate and coculture conditions and with the nuclei counterstained with Hoechst (blue); white arrows depict the lack of ATF4 and CHOP staining in Gfp-tagged *Bmpr1a^−/−^* cells, and white arrowheads depict colocalization of ATF4 and CHOP staining in WT cells. Scale bars, 50 μm for staining in separate cultures of WT and *Bmpr1a^−/−^*-Gfp cells and 100 μm for staining in cocultures. Bar graphs depict ATF4 (A) and CHOP (B) expressions as the percentage of pixel density in the images. (**C**) Cell competition assays between WT and *Bmpr1a^−/−^* cells and between WT and *Bmpr1a^−/−^*; *Atf4*^OE^ [OE (overexpressing)] cells. Bar graph represents the ratio between the final (d4) and initial cell numbers in separate and cocultures for each of these cell types. (**D**) Bar graph depicts the ratio between final (d4) and initial cell numbers of WT, *p53^−/−^,* WT, and *p53^−/−^*; *Atf4^−/−^* cells in separate and cocultures. *n* = 3 for all studies. Error bars denote SEM. *P < 0.05, **P < 0.01, ***P < 0.005, n.s., not significant; two-way ANOVA and Tukey’s post hoc test [(A) to (D)].

To establish the role of the ISR pathway during cell competition, we first analyzed the expression of ATF4 and CHOP in cocultured WT and *Bmpr1a^−/−^* ESCs. Immunofluorescence analysis revealed that, while in separate culture, the expression of ATF4 and CHOP was high in mutant cells and low in WT cells at day 3 of differentiation; this situation was reversed in coculture. Under this condition, the *Bmpr1a^−/−^* cells showed a down-regulation of ATF4 and CHOP expression, and the WT cells up-regulated the expression of these factors (Fig. 2, A and B). The decreased ISR expression found in *Bmpr1a^−/−^* cells in coculture suggests that this pathway may be regulating loser cell survival.

To test the above possibility, we did two things. First, we analyzed the impact of *Atf4* loss of function in ESCs and during differentiation to understand the effects of down-regulating this factor. We found that although *Atf4^−/−^* cells displayed normal proliferation when cultured under pluripotency conditions, their growth was severely compromised when induced to differentiate (fig. S2, C and D). This diminished proliferation was primarily due to apoptosis, as mutant cells cultured in N2B27 showed a marked increase in cleaved caspase 3 expression (fig. S2E), but no change in their cell cycle length, as inferred by the rate of 5-ethynyl-2’-deoxyuridine (EdU) incorporation (fig. S2F). We next overexpressed *Atf4* in *Bmpr1a^−/−^* cells under the control of a ubiquitous promoter [*Bmpr1a^−/−^; Atf4^OE^*; OE (overexpressing)]. Notably, we found that this was sufficient to completely rescue the elimination of mutant cells in coculture (Fig. 2C). This indicates that the ATF4 down-regulation observed in *Bmpr1a^−/−^* cells in coculture is likely causing their elimination. Given that we had also found an up-regulation of ATF4 in cocultured WT cells (Fig. 2, A and B), we tested the importance of ATF4 for winner cell behavior. For this, we resorted to a supercompetition model, where *p53^−/−^* eliminates WT cells (*10*), and we mutated *Atf4* in the *p53^−/−^* cells. We found that *p53^−/−^; Atf4^−/−^* cells no longer have a competitive advantage over WT cells, as they are no longer able to induce their elimination (Fig. 2D and fig. S2G). These results highlight the importance of ATF4 for determining how stressed and unstressed cells adapt to a heterogeneous environment. In the stressed cells, ATF4 down-regulation is driving their elimination, and in the unstressed cells, ATF4 up-regulation is supporting their competitive advantage. However, the question that then arises is how ATF4 plays these roles.

### Amino acid starvation activates the ISR to promote amino acid biosynthesis

**Fig. 3.**
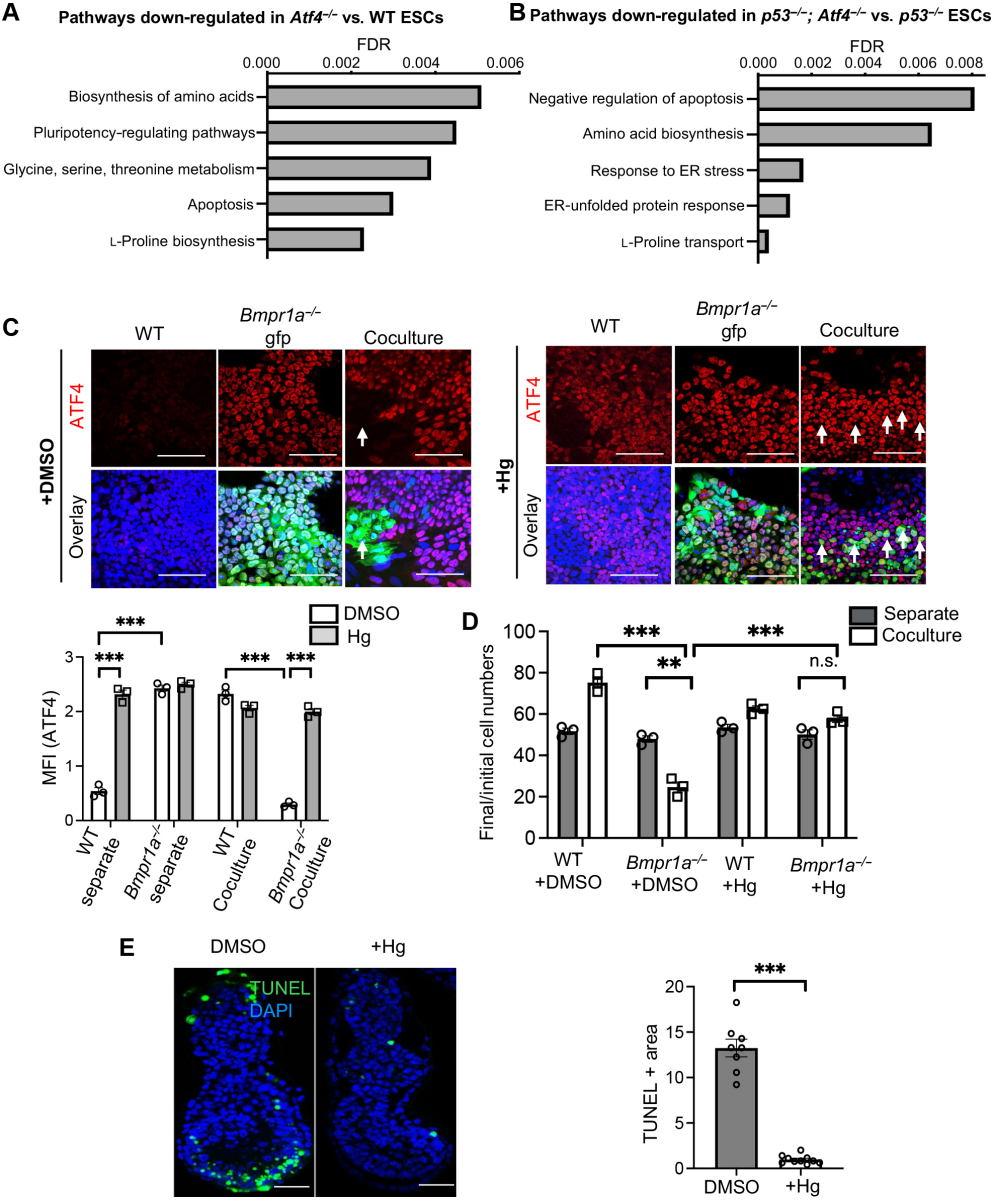
Activation of the amino acid deprivation branch of the ISR prevents cell competition. (**A** and **B**) Top down-regulated pathways identified by KEGG enrichment analysis of the transcriptomic datasets obtained from *Atf4^−/−^* versus WT cells (A) and p53^−/−^; *Atf4^−/−^* versus p53^−/−^ cells (B). FDR, false discovery rate. (**C**) Immunostaining analysis for ATF4 (red) in separate and cocultures of WT and *Bmpr1a^−/−^-*Gfp cells cultured for 48 hours in DMSO- or halofuginone (Hg; 20 nM)–containing media. Nuclei are counterstained with Hoechst (blue). Scale bars, 100 μm. Bar graph represents quantification of staining intensity of ATF4 (MFI in arbitrary units) in these cells. (**D**) Cell competition assays between WT and *Bmpr1a^−/−^* cells cultured for 48 hours in DMSO- or Hg-treated media depicted as the ratio of final (d3) cell numbers to the initial cell numbers of the cells in separate and cocultures. (**E**) Embryos (E5.5) were cultured for 18 hours in a medium supplemented with Hg (20 nM) or DMSO. Terminal deoxynucleotidyl transferase–mediated deoxyuridine triphosphate nick end labeling (TUNEL) assay (green) was performed to assess cell death in the epiblast cells of these embryos. Nuclei were counterstained with 4′,6-diamidino-2-phenylindole (DAPI; blue). Scale bars, 50 μm. Bar graph represents epiblast area stained positive for TUNEL as an indicator of cell death in epiblasts of the embryos. *n* = 3 for (A) and (D) and 8 for (B). Error bars denote SEM. For (C) and (D), **P < 0.01, ***P < 0.005, n.s., not significant; two-way ANOVA and Tukey’s post hoc test. For (E), ***P < 0.005, unpaired *t* test.

To address the mechanism by which ATF4 determines the outcome of cell competition, we performed transcriptomic comparisons of separate cultures of *Atf4^−/−^* versus WT cells and of *p53^−/−^; Atf4^−/−^* versus *p53^−/−^* cells at day 3 of differentiation. Differential cellular pathway analysis by Kyoto Encyclopedia of Genes and Genomes (KEGG) enrichment revealed that the topmost down-regulated pathways in both sets of cells with *Atf4* mutations were those associated with nonessential amino acid biosynthesis, transport, and metabolism (Fig. 3, A and B). This agrees with the finding that the ISR reroutes carbon utilization away from the tricarboxylic acid cycle and toward amino acid production (*24*). To further examine this possibility, we differentiated *Atf4^−/−^* ESCs in the presence of excess nonessential amino acids and found that this partially rescued the growth defect of mutant cells (fig. S2H). Testing individual or combinations of amino acids revealed that proline and asparagine could recapitulate the rescue seen with the pool of nonessential amino acids (fig. S2H). These results support the idea that the ISR is determining the outcome of cell competition by regulating amino acid levels.

One of the key drivers of ISR activation is amino acid starvation, as amino acid depletion triggers the sensor kinase general control non-derepressible 2 (GCN2) to phosphorylate eIF2α and activate the ATF4-CHOP axis (*20*, *21*). Given our finding that ATF4 primarily regulates amino acid biosynthesis in differentiating ESCs, one possibility is that defective cells activate the ISR in response to an amino acid deficiency caused by mitochondrial dysfunction. In this scenario, the down-regulation of the ISR in coculture could be due to these cells no longer sensing a lack of amino acids. Halofuginone (Hg) is a glutamyl-prolyl-tRNA synthetase inhibitor that induces the accumulation of uncharged prolyl tRNAs and activation of the amino acid starvation response (*25*). To test the above hypothesis, we differentiated WT, *Bmpr1a^−/−^*, and *Drp1^−/−^* cells separately or as WT/defective cocultures and treated them for 48 hours with Hg. We found that this increased ATF4 expression in all cell types and restored expression in *Bmpr1a^−/−^* and *Drp1^−/−^* cells to WT levels, both under separate and coculture conditions (Fig. 3C and fig. S3, A and B). This increase in ATF4 was sufficient to rescue defective cell elimination in coculture (Fig. 3D and fig. S3C). For Hg treatment, 1-, 5-, 10-, and 20-nM concentrations were tested, and only 20 nM produced a rescue (fig. S4, A and B). In the embryo, cell competition leads to a wave of cell death at E6.0 (*7*, *15*, *16*). We therefore examined the importance of activation of the ISR for this cell death, by culturing E5.5 embryos for 18 hours in the presence of Hg. We found that this abolished the cell death occurring at this stage in the embryo (Fig. 3E). A proportion of Hg-treated embryos presented epiblast malformations, suggesting that the amino acid starvation response may also be regulating postimplantation embryo morphogenesis. Together, these results indicate that activation of the amino acid starvation branch of the ISR with Hg can rescue loser cell elimination during cell competition. This points to the repression of the amino acid starvation branch of the ISR as a key step driving the elimination of defective cells in a competitive environment.

### Extracellular l-proline determines the outcome of cell competition

**Fig. 4.**
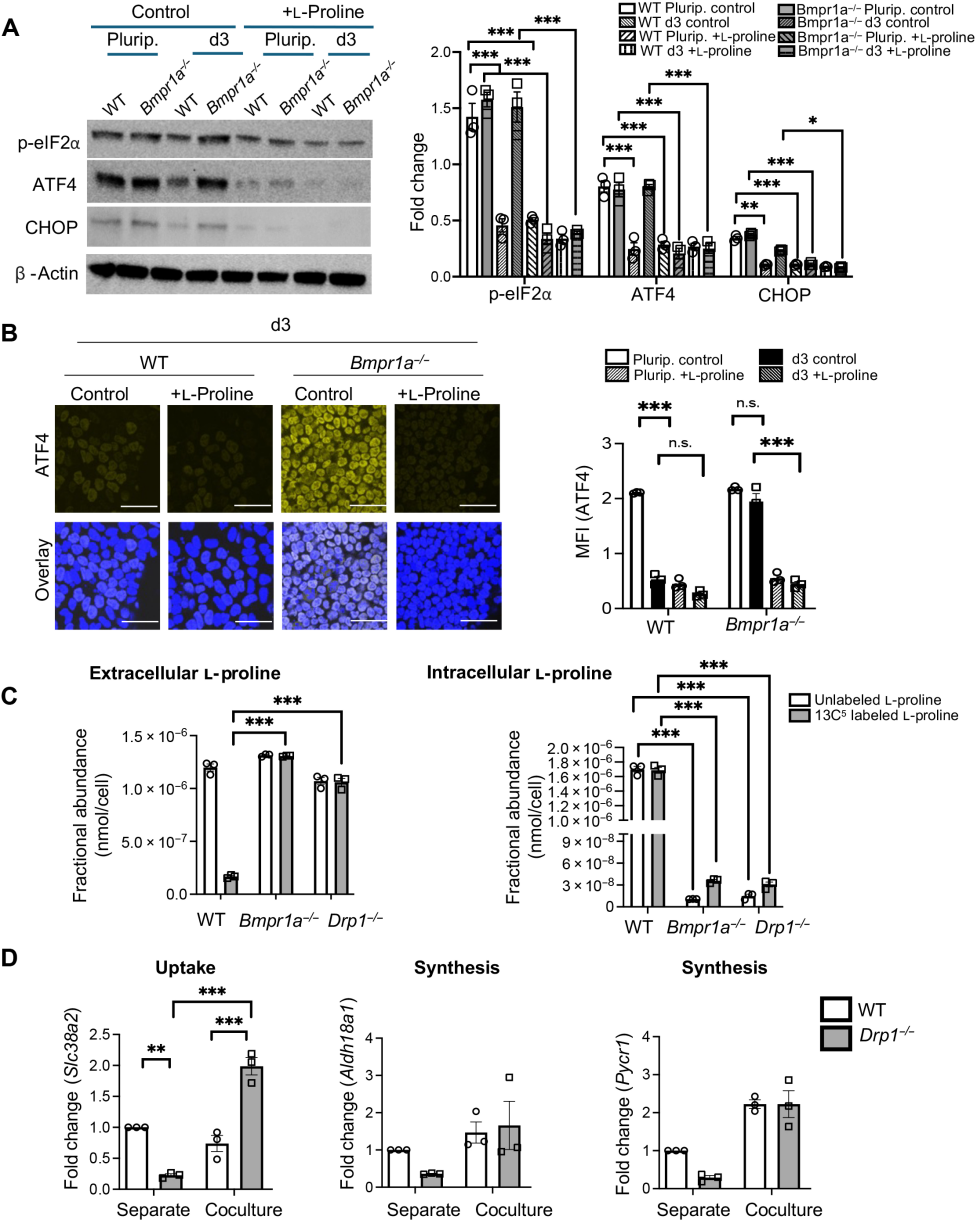
l-Proline represses ATF4-mediated ISR signaling. (**A**) WT and *Bmpr1a^− /−^* ESCs were cultured under Plurip. or induced to differentiate for d3 cultured in control medium or medium supplemented with l-proline for 48 hours (0.5 mM; +l-proline), and the expression of ISR proteins—p-eIF2α, ATF4, and CHOP—was analyzed by immunoblots. β-Actin served as a loading control. Bar graph represents fold change in expression of the proteins under various conditions. (**B**) Immunofluorescence analysis of ATF4 in WT and *Bmpr1a^−/−^* ESCs at d3 of differentiation cultured in control medium or medium supplemented with l-proline for 48 hours. Scale bars, 100 μm. Bar graph represents quantification of staining intensity of ATF4. (**C**) Bar graphs represent fractional abundance of extracellular and intracellular l-proline obtained by gas chromatography–mass spectrometry (GC-MS) of the medium samples and cell extracts from WT, *Bmpr1a^−/−^*, and *Drp1^−/−^* cells grown as homogeneous population at d3 of differentiation. (**D**) Quantitative reverse transcription polymerase chain reaction (RT-PCR) showing expression levels of the gene encoding neutral amino acid transporter responsible for l-proline uptake (*Slc38a2*) and of the genes encoding the enzymes that regulate l-proline synthesis (*Aldh18a1* and *Pycr1*) in separately and cocultured WT and *Drp1^−/−^* ESCs at d3 of differentiation. Gene expression is normalized against *Gapdh*. *n* = 3 for all studies. Error bars denote SEM. *P < 0.05, **P < 0.01, ***P < 0.005, n.s., not significant; two-way ANOVA and Tukey’s post hoc test.

The finding that defective cells no longer sense amino acid deprivation in coculture suggests that under this condition, they are exposed to increased levels of one or several amino acids. To test this possibility, we first set out to establish which amino acids repressed the ISR during cell competition. Our transcriptional profiling of Atf4 mutant cells suggested that this factor regulates l-proline biosynthesis and transport (Fig. 3, A and B, and fig. S2H), an amino acid previously shown to repress ATF4 in ESCs (*26*). We therefore first analyzed the ability of l-proline to repress the ISR by treating WT, *Bmpr1a^−/−^*, and *Drp1^−/−^* ESCs, cultured separately under pluripotency and differentiation conditions, for 48 hours with excess l-proline. We found that l-proline induced a down-regulation of p-eIF2α, ATF4, and CHOP in all cell types and under all conditions analyzed (Fig. 4, A and B, and fig. S5, A and B). Furthermore, we also observed that l-proline treatment induced cell death, as measured by cleaved caspase 8 levels, in WT, *Bmpr1a^−/−^*, and *Drp1^−/−^* cells when induced to differentiate (fig. S5C). In contrast, other amino acids were unable to repress the ISR (fig. S6). These results suggest that l-proline is uniquely capable of repressing the ISR, and it may be inducing the ISR down-regulation that causes defective cell elimination in coculture.

Next, we analyzed l-proline metabolism in WT, *Bmpr1a^−/−^*, and *Drp1^−/−^* cells. For this, we first separately cultured these cells in l-proline–deficient medium supplemented with labeled l-proline (^13^C_5_–l-proline). We replaced the cells with fresh labeled l-proline–containing medium every 24 hours from day 0 of differentiation and collected both the medium and cells for metabolite extraction at day 3 of differentiation. This allows analysis of l-proline synthesis, by measuring the presence of unlabeled l-proline, as well as its uptake, by studying the extracellular versus intracellular localization of ^13^C5–labeled l-proline. Gas chromatography–mass spectrometry (GC-MS) analysis of supernatants indicated that at day 3 of differentiation, both WT and defective cells had similar levels of extracellular unlabeled l-proline, indicating that the three cell types had a similar capacity to secrete this amino acid. In contrast to this, when we analyzed labeled l-proline levels, we found that while *Bmpr1a^−/−^* and *Drp1^−/−^* supernatants showed higher l-proline levels than WT cells, their cell pellets displayed significantly lower levels (Fig. 4C). The high and low intracellular levels of l-proline in *Bmpr1a^−/−^* and *Drp1^−/−^* cells indicate that these cells have a deficiency in l-proline uptake. When we analyzed the levels of unlabeled l-proline in cell pellets, we found that mutant pellets also had lower levels than those from WT cells (Fig. 4C). The lower levels of intracellular, newly synthesized/unlabeled l-proline, signifies that *Bmpr1a^−/−^* and *Drp1^−/−^* cells also likely have defective l-proline synthesis. Given that l-proline represses ATF4 (Fig. 4, A and B, and fig. S5, A and B), the lower l-proline uptake and synthesis observed in mutant cells explain the elevated ATF4 expression seen in these cells in separate cultures at day 3 of differentiation (Fig. 1, E and F).

To understand the causes for the differing ability of WT and mutant cells to take up and synthesize l-proline, we analyzed in WT and *Drp1^−/−^* cells the expression levels of *Slc38a2*, the gene that encodes the neutral amino acid transporter responsible for l-proline uptake and of *Aldh18a1* [encoding delta-1-pyrroline-5-carboxylate synthetase (P5CS)] and *Pycr1*, that regulate de novo l-proline synthesis. In agreement with the lower intracellular levels of l-proline observed in defective cells, we found that in separate cultures, *Drp1^−/−^* cells show a down-regulation in the expression of both the genes responsible for l-proline uptake and its synthesis (Fig. 4D). In contrast to this, when we analyzed the expression of these factors in coculture, we found that *Aldh18a1* and *Pycr1* were up-regulated in mutant cells to the levels found in WT cells, and *Slc38a2* was increased in *Drp1^−/−^* cells to threefold higher levels than those of WTs (Fig. 4D). This suggests that the presence of WT cells has induced mutant cells to increase l-proline uptake and synthesis. This finding correlates with the repression of ATF4 expression that we see in mutant cells in coculture (Fig. 2A) and provides a likely explanation for their elimination.

**Fig. 5.**
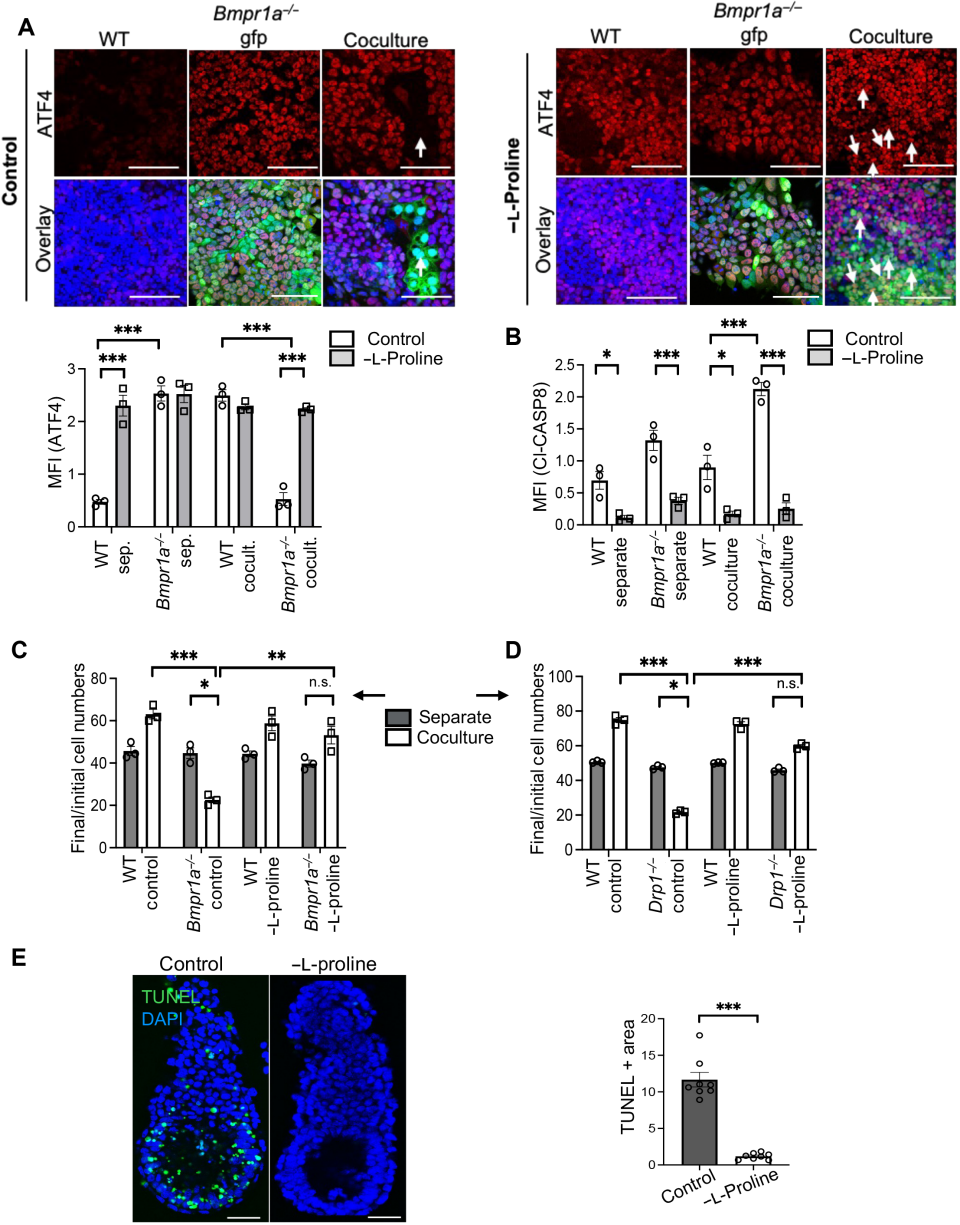
l-Proline–mediated regulation of ATF4 levels leads to competitive loser cell elimination. (**A**) Immunofluorescence analysis for ATF4 (red) in separate and cocultures of WT and *Bmpr1a^−/−^* cells, cultured in control and l-proline–deficient media (−l-proline media). Nuclei are counterstained with Hoechst (blue). Scale bars, 100 μm. Bar graphs represent quantification of staining intensity of ATF4. (**B**) Bar graph represents quantification of staining intensity of cleaved Caspase 8 in separate and cocultures of WT and *Bmpr1a^−/−^* cells, cultured in control and L-proline–deficient media. sep., separate; cocult., coculture. (**C** and **D**) Bar graphs representing the ratio of final (d3) to initial cell numbers of WT, *Bmpr1a^−/−^* (C), WT, and *Drp1^−/−^* cells (D) in control and L-proline–deficient media for separate and cocultures. (**E**) Embryos (E5.5) were cultured for 18 hours in control (N2B27) and –L-proline–deficient media. TUNEL assay (green) was performed to assess cell death in epiblast cells of the embryos. Nuclei counterstained with DAPI (blue). Scale bars, 50 μm. Bar graph represents epiblast area stained positive for TUNEL as an indicator of cell death in epiblasts of the embryos. *n* = 3 for (A) to (D) and 8 for (E)*.* Error bars denote SEM. *P < 0.05, **P < 0.01, ***P < 0.005, n.s., not significant; two-way ANOVA and Tukey’s post hoc test.

The above results suggest that the uptake of extracellular l-proline may determine the outcome of cell competition, with the increased ability of defective cells to do so in coculture compared to separate cultures driving their elimination. To test this possibility, we performed our cell competition assays in media without l-proline (−l-proline). When either *Bmpr1a^−/−^* or *Drp1^−/−^* cells were cocultured with WT cells in l-proline–depleted media, we found that the lack of exogenous l-proline led to a marked increase in ATF4 expression in both defective cell types (Fig. 5A and fig. S7, A and B). Notably, the levels of ATF4 expression in cocultured defective cells were now equivalent to those found in these cells in separate cultures. l-Proline–deficient media also increased ATF4 expression in WT cells in separate cultures but not in coculture, meaning that under this condition, WT and defective cells now have the same levels of ATF4 expression. The increase in ATF4 expression in defective cells was accompanied by a decrease in apoptosis, as measured by the levels of cleaved caspase 8 (Fig. 5B and fig. S8, A and B). The coculture of defective cells with WT cells in media lacking l-proline completely rescued their elimination (Fig. 5, C and D). Notably, this rescue was not due to a block in differentiation, as l-proline–deficient media did not impair the ability of ESCs to differentiate (fig. S9A), and adding back half the normal amount of l-proline from day 1 of differentiation restored the competitive ability of the cells (fig. S9, B and C). Last, when we tested the effects of depletion of arginine, asparagine, glutamate, lysine, phenylalanine, tryptophan, alanine, serine, glycine, or methionine, none of these prevented the elimination of *Bmpr1a^−/−^* or *Drp1^−/−^* cells in coculture (fig. S10).

We next tested the in vivo relevance of this observation. When E5.5 embryos were cultured in media lacking l-proline for 18 hours, we found that this was sufficient to abolish the cell death occurring in the epiblast (Fig. 5E). Therefore, the levels of extracellular l-proline determine the outcome of cell competition both in cells and the embryo. Collectively, these results indicate that during cell competition, defective cells increase l-proline uptake from the extracellular environment. This increases intracellular l-proline levels and induces their elimination by repressing the prosurvival ISR. This highlights how mitochondrial dysfunction sensitizes cells to their metabolic environment and suggests that this mechanism ensures that the epiblast is composed solely of the fittest cells during the early stages of postimplantation development.

## DISCUSSION

Cell competition has been shown to regulate cell fitness in a wide range of contexts, from the developing embryo to the aging tissue (*3*, *6*, *27*). An important role of cell competition is to preserve tissue function by eliminating less-fit cells. A key feature of this process is that cells need to be able to measure and respond to the fitness levels of neighboring cells. However, how a cell can sense the fitness of another is still poorly understood, with both mechanical and chemical signals potentially being involved (*6*).

Here, we approached this problem by studying the fate of cells with mitochondrial dysfunction, the predominant type of defective cell eliminated by cell competition during early mouse development (*17*). We find that during the first steps of pluripotent stem cell differentiation, mitochondrial dysfunction leads to activation of the ISR, a pathway that acts to restore cellular homeostasis in response to diverse stress stimuli (*20*, *21*). Both in the embryo and during pluripotent stem cell differentiation, these dysfunctional cells require the ISR for their survival, making them susceptible to repression of this pathway. We show that in a heterogeneous environment, WT cells exploit this vulnerability and induce defective cell elimination via repression of the ISR and its key effector, ATF4. This process allows for cells with robust mitochondrial activity to maximize their colonization of the tissue and potentially optimize tissue function.

We also find that when there is a high cell density, defective cells avoid elimination. This suggests that when high numbers of cells are damaged, activation of the ISR would allow them to evade cell competition. Similar formation of a protective environment by increased numbers of loser cells has also been found to occur in the *Drosophila* imaginal wing disc (*28*). The increased survival of defective cells as tissue crowding increases argues that chemical signals rather than mechanical stresses likely determine the outcome of the cell competition occurring during differentiation.

**Fig. 6.**
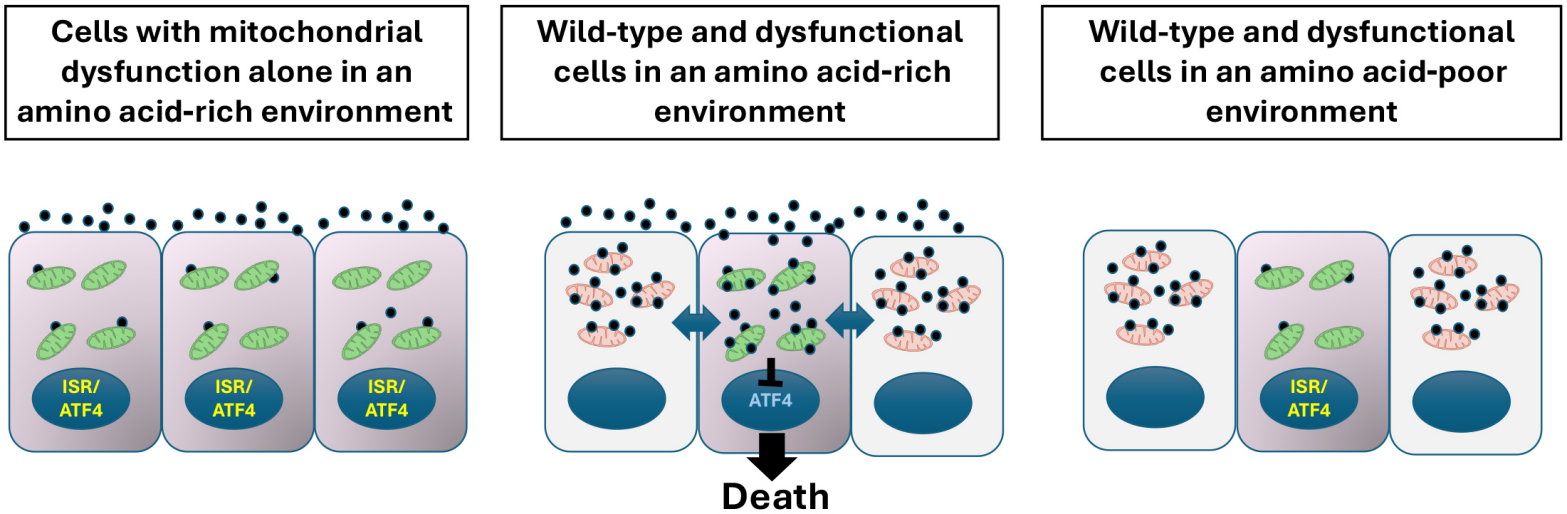
Model for defective cell elimination during cell competition. Diagrammatic representation showing how when cells with mitochondrial dysfunction are in a homotypic environment, they have low intracellular levels of l-proline (black circles) and ISR activation (**left**). In a competitive environment, the presence of WT cells induces increased l-proline metabolism in the dysfunctional cells, repressing the ISR and causing their elimination (**middle**). In contrast to this, low levels of extracellular amino acids reduce l-proline uptake in the cells with mitochondrial dysfunction, restoring ISR signaling and allowing for their survival (**right**).

However, what chemical signals could regulate this cell competition? During the onset of differentiation, ESCs switch from relying on the uptake via macropinocytosis and digestion of exogenous proteins as a source of amino acids to the direct uptake of these metabolites (*29*). Notably, the exit of naïve pluripotency is required for competition in ESCs (*15*). In consonance with these observations, we find a central role for the amino acid l-proline in cell competition. In mouse ESCs, l-proline has been suggested to play differing roles, from inducing the proliferation of naïve cells and allowing these cells to adopt an early primed pluripotent state (*30*, *31*) to driving differentiation to a primitive ectoderm state (*32*, *33*). In contrast to these observations, we show that during the onset of differentiation, l-proline induces the competitive elimination of cells with mitochondrial dysfunction. This is supported by several lines of evidence. First, in agreement with previous findings (*26*), we identify that l-proline represses the amino acid starvation response branch of the ISR. Given that, during differentiation, the ISR is required for the survival of cells with mitochondrial dysfunction, this repression would be sufficient to induce dysfunctional cell elimination. Second, we find that in coculture, WT cells induce an increase in the expression of the enzymes that metabolize and import l-proline in cells with mitochondrial dysfunction. This indicates that in a competitive environment, these dysfunctional cells will experience an increase in l-proline metabolism, which correlates with the repression of the ISR that these cells show. Last, we demonstrate that removing l-proline from the culture media of ESCs or mouse embryos is sufficient to restore ISR signaling in defective cells and prevents their elimination by cell competition. These results imply that rather than sensing the fitness of neighboring cells, during cell competition, WT cells overload defective cells with a metabolite that is toxic to them (Fig. 6).

The mechanism of cell competition that we describe here does not necessitate the sensing of relative fitness levels but, instead, is caused by the relative susceptibility to extracellular signals. We propose that WT cells secrete signals, either growth factors or metabolites, that promote l-proline metabolism in their neighbors. These signals could potentially be acting to coordinate growth but would have a negative impact on cells with mitochondrial dysfunction, as they would repress the ISR in these cells, inducing their elimination. Two other pathways have been implicated in fitness sensing in *Drosophila* and cancer cells: differential expression of FLOWER isoforms (*34*, *35*) and activation of the innate immune pathway (*36*). Viral infection, which can activate the innate immune pathway, is also a trigger for the ISR (*20*, *21*). Similarly, FLOWER can act as a calcium channel, and changes in calcium levels can lead to unfolded proteins and ER stress, another trigger of the ISR. Furthermore, in *Drosophila*, autocrine glutamate signaling via the *N*-methyl-d-aspartate receptor is required for the outcompetition of loser cells (*37*, *38*). l-Proline catabolism generates glutamate, suggesting a role for l-proline metabolism here as well. It will be interesting in the future to investigate any possible cross-talk between these pathways.

Last, our results also have implications for the understanding of how nutrient availability in the extracellular environment shapes tissue composition and function. Our finding that l-proline–deficient medium abolishes the negative selective pressure acting on defective cells is counterintuitive, as it suggests that the survival of dysfunctional cells is enhanced under nutrient-poor conditions. These results are in accordance with our observation that defective cell elimination is rescued at higher cell densities. A likely explanation for this finding is that at higher cell densities, the levels of extracellular l-proline fall below the critical threshold required for cell competition due to the accelerated depletion of nutrients that occurs in confluent cultures. The hypothesis that nutrient deprivation suppresses cell competition is supported by one of the earliest findings of the cell competition field, which, in fly, starvation prevents the competitive elimination of ribosomal mutant *Minute* clones from the *Drosophila* imaginal wing disc (*39*). What these results imply is that cell competition optimizes tissue function in response to nutrient availability, allowing for replacement of less-fit cells to take place in nutrient-rich environments but not under conditions of nutrient scarcity. Our work shows that amino acid sensing, and activation of the amino acid starvation response, plays a central role in the response to low nutrients, suggesting that protein levels in the diet could determine the nature of the competitive interactions occurring in the tissue. We propose that this represents a previously unidentified tissue-sparing mechanism that could offer protection, for instance, in cases of maternal malnutrition or low-protein diets. However, this protection may come at the expense of optimal tissue function, as it allows the persistence of dysfunctional cells within the tissue.

## MATERIALS AND METHODS

### Cell lines and cell culture

E14, provided by A. Smith, from Cambridge University, was used as WT control cells, either tdTomato-labeled or unlabeled. GFP-labeled cells defective in BMP signaling (*Bmpr1a^−/−^*), cells null for *Drp1^−/−^*, and *p53^−/−^* ESCs are described elsewhere (*10*, *15*–*17*). *Atf4^−/−^* and *p53^−/−^*; *Atf4^−/−^* ESCs were generated by CRISPR mutagenesis as previously described (*16*).

Cells were cultured at 37°C and 5% CO_2_ in Glasgow's minimal essential media (GMEM) supplemented with 10% fetal bovine serum (FBS), 2 mM glutamine, 1× MEM nonessential amino acids, 1 mM sodium pyruvate, 0.1 mM β-mercaptoethanol (all Thermo Fisher Scientific), and 100 *U*/ml leukemia inhibitory factor (generated and tested in the laboratory). The cells were grown in a monolayer, with the medium changed daily, and passaged every 2 days using trypsin-EDTA (Sigma-Aldrich) for dissociation. Cell lines were routinely tested for mycoplasma contamination.

### ESC differentiation and cell competition assays

To induce differentiation, mouse ESCs were seeded at low density (8x10^4^ cells/ml for 12-well plates and 1.6x10^5^ cells/ml for 6-well plates) on fibronectin-coated plates (Merck) in N2B27 medium: an equimolar mix of Dulbecco’s modified Eagle’s minimum essential medium (DMEM)/F-12 and Neurobasal media, containing 0.5× N2, 0.5× B27, 2 mM l-glutamine, and 0.1 mM 2-mercaptoethanol (all from Thermo Fisher Scientific). The medium was changed daily, allowing differentiation to proceed for 3 to 4 days. All treatments began 24 hours after cell seeding and lasted for 48 hours. For l-proline treatment (+l-proline), cells were cultured in N2B27 medium with the addition of 0.5 mM l-proline. For treatment with different amino acids (+Arg, +Asn, +Glu, +Lys, +Phe, +Trp, Ala, +Cys, +Gly, +Met, and +Ser), cells were administered with the amino acids (0.5 mM for 48 hours) under the pluripotent (ESC) and differentiating conditions (d3). For Hg (Selleckchem) treatment, 1-, 5-, 10-, and 20-nM concentrations were tested, and only 20 nM produced a rescue. A total of 20 nM of Hg dissolved in DMSO was supplemented to the N2B27 medium.

DMSO served as vehicle control. A total of 1-, 5-, 10-, and 20-nM concentrations were tested, and only 20 nM produced a rescue. For pan-CI treatment (Z-VAD-FMK, R&D Systems), differentiating cells were treated with 100 μM inhibitor, with DMSO as vehicle control. l-Proline–deficient medium was prepared by manually dissolving amino acids (table S1), except l-proline, in water-based neuronal cell culture medium without amino acids (US Biological Life Sciences). Media deficient in other amino acids (−Arg, −Asn, −Glu, −Lys, -Phe, −Trp, −Ala, −Cys, −Gly, −Met, and −Ser) were prepared in a similar fashion, omitting the respective amino acid while adding all other amino acids.

For cell competition assays, cells were seeded onto 12-well plates coated with fibronectin (Merck) at a density of 8 × 10^4^ cells per well for separate cultures and 4 × 10^4^ cells of each cell type at a 50:50 ratio per well for cocultures. The cells were then cultured in differentiation medium. On days 3 and 4 of differentiation, cells were dissociated with trypsin-EDTA, and cell counting was performed using the Countess 3 FL (Thermo Fisher Scientific), following the manufacturer’s instructions. Cells were resuspended in 3% FBS for fluorescence-activated cell sorting (FACS). The proportions of each cell type in cocultures were determined using an LSR II Flow Cytometer (BD Bioscience), based on the fluorescent tag of the ubiquitously expressed GFP or tdTomato in one of the cell populations.

### Mice and embryo culture

All mice were maintained on a 10-hour–14-hour light-dark cycle and treated in accordance with the Home Office’s Animals (Scientific Procedures) Act 1986. WT mice analyzed were maintained on a CD1 outbred genetic background. Noon of the day of finding, a vaginal plug was designated E0.5. Embryo dissection was performed in M2 medium (Sigma-Aldrich). No distinction was made between male and female embryos during the analysis. All animal experiments were approved by the appropriate ethics committee (Home Office Project License_PP6959042). For the CI, Hg, and –l-proline treatments, embryos were dissected at E5.5, cultured overnight (18 hours) at 37°C and 5% CO_2_ in 200 μM Z-VAD-FMK, 20 nM Hg, or the equivalent DMSO volume in N2B27 medium [Neurobasal media; DMEM F12 media; 0.5× B27 supplement; 0.5× N-2 supplement; 0.1 mM 2-mercaptoethanol; and 2 mM glutamine (all Thermo Fisher Scientific)], and fixed. For the –l-proline experiments, embryos were cultured for 18 hours in either –l-proline or control medium (N2B27 medium) and then fixed. After fixation, embryos were processed for immunofluorescence analysis of cell death by terminal deoxynucleotidyl transferase–mediated deoxyuridine triphosphate nick end labeling (TUNEL) staining. TUNEL staining was performed using In Situ Cell Death Detection Kit (Roche) following the manufacturer’s protocol, as previously described (*16*).

### Immunoblotting

Cell lysates were collected using radioimmunoprecipitation assay (RIPA) buffer [50 mM tris-HCl at pH 6.8, 1% sodium deoxycholate 150 mM sodium chloride, 1% NP-40, 1 mM EDTA, 1× phosphatase inhibitor (Roche), and 1× protease inhibitor (Roche)], quantified using the bicinchoninic acid (BCA) assay quantification (Thermo Fisher Scientific), and resolved using Criterion XT precast gels (Bio-Rad), followed by transfer to polyvinylidene difluoride membranes. Antibodies used are as follows: p-eIF2α (1:1000), rabbit polyclonal [Cell Signaling Technology (CST), 9721]; ATF4 (1:1000), rabbit monoclonal (CST, 11,815), CHOP (1:500), mouse monoclonal (sc-7351), cleaved caspase 3 (1:1000), rabbit polyclonal (SCT, 9661), proliferating cell nuclear antigen (PCNA; 1:5000), mouse monoclonal (sc-56), β-actin (1:2500), and rabbit polyclonal (CST, 4967). Primary antibody incubation was performed overnight at 4°C. This was followed by three tris-buffered saline with tween 2 (TBST) washes and secondary antibody incubation [anti-mouse (CST, 7076) or anti-rabbit (CST, 7074) conjugated to horseradish peroxidase (1:4000)] for 1.5 hours at room temperature. Blots were developed using enhanced chemiluminescence reagents (Merck) and imaged with the ChemiDoc MP system (Bio-Rad). To reprobe the membrane for alternative proteins, blots were stripped with stripping buffer (0.005% EDTA) by heating for 5 min. Protein expression analysis by densitometry was performed using ImageJ Fiji software and normalized to loading controls, β-actin or PCNA.

### Immunofluorescence

Cells were fixed in 4% paraformaldehyde (PFA) for 10 min, permeabilized with 0.3% Triton X-100 (0.1% for cleaved caspase 3) for 5 min, and blocked with 3% bovine serum albumin (Sigma-Aldrich) and 0.3% Triton X-100 (0.1% for cleaved Caspase 3) for 1 hour. Staining with primary antibodies [ATF4 (1:200), mouse monoclonal (sc-390063), CHOP (1:200), mouse monoclonal (CST, 2895), cleaved caspase 8 (1:100), and rabbit monoclonal (CST, 8592)] was performed overnight at 4°C in blocking buffer. After three washes in phosphate-buffered saline (PBS), secondary antibodies (Alexa Fluor 647 and Thermo Fisher Scientific; 1:500 dilution) and Hoechst (Thermo Fisher Scientific; 1:1000 dilution) were incubated for 1 hour. Coverslips were then mounted on glass slides using VECTASHIELD antifade mounting medium (VectorLabs).

Embryos were fixed using 4% PFA in PBS + 0.01% Triton X-100 and 0.1% Tween. Permeabilization was performed with 0.5% Triton X-100 in PBS for 20 min, and embryos were blocked using 2% horse serum in PBS + 0.1% Triton X-100 (PBT) for 45 min. Primary antibodies, as above, were incubated with embryos in blocking solution overnight at 4°C. Following three washes in PBT, the embryos were incubated with secondary antibodies and 4′,6-diamidino-2-phenylindole (DAPI), as above, for 1 hour at 4°C. TUNEL staining was performed using In Situ Cell Death Detection Kit (Roche) following the manufacturer’s protocol, as previously described (*16*). Embryos were imaged in embryo dishes (Nunc) in a drop of VECTASHIELD. All images were captured on a Zeiss LSM 780 confocal microscope (40× oil objective lens for cells and 20× objective lens for embryos) and analyzed using ImageJ Fiji software (*40*).

### Proliferation assays

Cells were incubated with EdU from the Click-iT kit (Thermo Fisher Scientific) for 2 hours according to the manufacturer’s instructions. Cells were then detached from plates using Accutase and analyzed by flow cytometry using the LSR II Flow Cytometer and FlowJo software.

### Bulk RNA sequencing

Cells grown for 3 days in N2B27 medium were recovered into growth media and then resuspended in RLT lysis buffer (QIAGEN). RNA extraction was performed using the QIAGEN RNeasy kit according to the manufacturer’s instructions. Quality control, library preparation, and sequencing were performed by the BRC Genomics Center. RNA samples were quantified using a Qubit fluorometer (Thermo Fisher Scientific), and the quality was assessed by TapeStation electrophoresis (Agilent). mRNA was isolated using oligo(dT) beads. mRNA was then fragmented, converted to cDNA, and ligated to Illumina adapters. Following sample indexing, the quality of cDNA libraries was also assessed by TapeStation. Sequencing was performed using the NextSeq 2000 system (Illumina). Sequencing reads were aligned to the mouse genome (mm9) using TopHat2 (*41*), and differential expression (DE) was analyzed using the DESeq2 package (*42*). The enrichment analysis for the bulk RNA sequencing (RNA-seq) datasets was performed using the g:Profiler tool76. The list of up-regulated, down-regulated, and background genes related to the DE analysis for the bulk RNA-seq dataset is provided in table S3.

### RNA extraction and quantitative reverse transcription polymerase chain reaction

RNA was extracted with the RNeasy mini kit (QIAGEN), and SuperScript III reverse transcriptase (Thermo Fisher Scientific) was used for cDNA synthesis according to the manufacturer’s instructions. Quantitative reverse transcription polymerase chain reaction (RT-PCR) was performed by amplification with LightCycler 480 SYBR Green Master (Roche). The primers used are listed. RNA samples were collected from three independent experiments.

### Cell sorting

E14 tdTomato and *Drp1^−/−^* cells were cultured as separate and cocultures as mentioned above and then subjected to FACS using a BD FACSAria II sorter, followed by RNA extraction, cDNA synthesis, and quantitative polymerase chain reaction analysis. For FACS, cells were trypsinized and resuspended in 500 ml of 3% FBS in PBS. Cell suspensions were passed through a 40-mm nylon mesh filter right before sorting. Postsorting, cells were collected in sort buffer [PBS, 1 mM EDTA, 25 mM Hepes (pH 7.0), and 1% FBS] and subjected immediately to RNA extraction as per the method mentioned above.

### Metabolomic analysis

For ESCs and differentiated samples, cells were cultured in –l-proline medium supplemented with 0.21 mM labeled l-proline [^13^C_5_–l-proline (Cambridge Isotope Laboratories), provided by K. Vousden’s laboratory]. Levels of labeled and unlabeled l-proline were determined in cell culture supernatants and cell extracts.

At the time of harvest, medium samples were first collected from each well, and cells were washed with ice-cold PBS and scraped out in ice-cold high-performance liquid chromatography (HPLC)–grade methanol containing 1 nmol of norleucine (Merck, N8513) as an internal standard. The cell suspensions were collected in prechilled 2-ml Eppendorf tubes and mixed with an equal volume of HPLC-grade chloroform and GC-MS–grade water. The mixtures were then centrifuged at 14,800*g* for 10 min at 4°C to achieve biphasic partitioning. The polar metabolites in the upper phase of the samples were collected, dried, and resuspended in 100 μl of methanol containing *scyllo*-inositol (Merck, I8132) at 1 nmol per sample. The polar metabolite extracts were then transferred to a GC-MS vial with insert. The samples were dried in a SpeedVac and washed twice with methanol.

For metabolite extraction from culture supernatants/medium, 5 μl of media per sample was added to a GC-MS vial with insert. A total of 10 μl of 0.1 mM *scyllo*-inositol (internal standard) was added to the vials, followed by drying in SpeedVac (~15 min) and washing twice with methanol. A total of 5 μl of metabolite mix standards containing 1 nmol of *scyllo*-inositol were prepared in separate vials as mentioned above.

Data acquisition was performed largely as previously described (*43*) using an Agilent 7890B-5977A GC-MSD in electron ionization (EI) mode after derivatization of twice methanol-washed, dried polar extracts by addition of (i) 20 μl of methoxyamine hydrochloride [20 mg/ml in pyridine (both from Sigma-Aldrich) at room temperature overnight (>16 hours)] and (ii) 20 μl of *N*,*O*-Bis(trimethylsilyl)trifluoroacetamide containing 1% trimethylchlorosilane (Sigma-Aldrich) at room temperature for at least 1 hour. GC-MS parameters for polar analyses were as follows: carrier gas (helium); flow rate (0.9 ml/min); column (Agilent, DB-5MS); inlet (270°C); temperature gradient (70°C for 2 min), ramp to 295°C (12.5°C/min), and ramp to 320°C (25°C/min, 3-min hold). The scan range was a mass/charge ratio of 50-550 (polar). Data were acquired using MassHunter software (version B.07.02.1938). Data analysis was performed using Manic software, an in-house–developed adaptation of the GAVIN package (PMID: 21575589). Metabolites were identified and quantified by comparison to authentic standards, and label incorporation was estimated as the percentage of the metabolite pool containing one or more ^13^C atoms after correction for natural abundance. In addition, we normalized peak areas to the cell number of the samples and internal standard *scyllo*-inositol.

### Quantification and statistical analysis

Flow cytometry data were analyzed with FlowJo software. Western blot quantification was performed using ImageJ software. Protein expression levels were normalized to loading controls—β-actin or PCNA. Statistical analysis was performed using GraphPad Prism v8.0.0 software. Statistical methods used are indicated in the relevant figure legends. For all in vitro analysis experiments, we used a sample size of three independent biological replicates, and for ex vivo embryo experiments, we used a sample size of eight biological replicates. Statistical significance was considered with a confidence interval of 0.05%; *P < 0.05, **P < 0.01, and ***P < 0.005. Data obtained from cell competition assays, Western blots, and immunofluorescence quantification (multiple-group comparisons) were analyzed by two-way analysis of variance (ANOVA), followed by Tukey’s multiple-comparison test. Data obtained from Western blot, immunostaining analysis for embryos, and proliferation assay were analyzed by unpaired *t* test.

## References

[1] G. Morata, Cell competition: A historical perspective. Dev. Biol. 476, 33–40 (2021). 33775694 10.1016/j.ydbio.2021.02.012

[2] J. Nichols, A. Lima, T. A. Rodríguez, Cell competition and the regulative nature of early mammalian development. Cell Stem Cell 29, 1018–1030 (2022). 35803224 10.1016/j.stem.2022.06.003

[3] E. Madan, R. Gogna, E. Moreno, Cell competition in development: Information from flies and vertebrates. Curr. Opin. Cell Biol. 55, 150–157 (2018). 30208354 10.1016/j.ceb.2018.08.002

[4] L. Esteban-Martínez, M. Torres, Metabolic regulation of cell competition. Dev. Biol. 475, 30–36 (2021). 33652024 10.1016/j.ydbio.2021.02.011

[5] N. E. Baker, Emerging mechanisms of cell competition. Nat. Rev. Genet. 21, 683–697 (2020). 32778819 10.1038/s41576-020-0262-8 PMC8205513

[6] S. Bowling, K. Lawlor, T. A. Rodriguez, Cell competition: The winners and losers of fitness selection. Development 146, dev167486 (2019). 31278123 10.1242/dev.167486

[7] C. Clavería, G. Giovinazzo, R. Sierra, M. Torres, Myc-driven endogenous cell competition in the early mammalian embryo. Nature 500, 39–44 (2013). 23842495 10.1038/nature12389

[8] E. Moreno, K. Basler, dMyc transforms cells into super-competitors. Cell 117, 117–129 (2004). 15066287 10.1016/s0092-8674(04)00262-4

[9] C. de la Cova, M. Abril, P. Bellosta, P. Gallant, L. A. Johnston, *Drosophila* myc regulates organ size by inducing cell competition. Cell 117, 107–116 (2004). 15066286 10.1016/s0092-8674(04)00214-4

[10] S. Perez Montero, P. K. Paul, A. di Gregorio, S. Bowling, S. Shepherd, N. J. Fernandes, A. Lima, R. Pérez-Carrasco, T. A. Rodriguez, Mutation of p53 increases the competitive ability of pluripotent stem cells. Development 151, dev202503 (2024). 38131530 10.1242/dev.202503 PMC10820806

[11] J. A. Valverde-Lopez, L. Li-Bao, R. Sierra, E. Santos, G. Giovinazzo, C. Díaz-Díaz, M. Torres, P53 and BCL-2 family proteins PUMA and NOXA define competitive fitness in pluripotent cell competition. PLOS Genet. 20, e1011193 (2024). 38489392 10.1371/journal.pgen.1011193 PMC10971546

[12] L. Wagstaff, M. Goschorska, K. Kozyrska, G. Duclos, I. Kucinski, A. Chessel, L. Hampton-O’Neil, C. R. Bradshaw, G. E. Allen, E. L. Rawlins, P. Silberzan, R. E. Carazo Salas, E. Piddini, Mechanical cell competition kills cells via induction of lethal p53 levels. Nat. Commun. 7, 11373 (2016). 27109213 10.1038/ncomms11373 PMC4848481

[13] C. J. Price, D. Stavish, P. J. Gokhale, B. A. Stevenson, S. Sargeant, J. Lacey, T. A. Rodriguez, I. Barbaric, Genetically variant human pluripotent stem cells selectively eliminate wild-type counterparts through YAP-mediated cell competition. Dev. Cell 56, 2455–2470.e10 (2021). 34407428 10.1016/j.devcel.2021.07.019 PMC8443275

[14] M. Vishwakarma, E. Piddini, Outcompeting cancer. Nat. Rev. Cancer 20, 187–198 (2020). 31932757 10.1038/s41568-019-0231-8

[15] M. Sancho, A. Di-Gregorio, N. George, S. Pozzi, J. M. Sánchez, B. Pernaute, T. A. Rodríguez, Competitive interactions eliminate unfit embryonic stem cells at the onset of differentiation. Dev. Cell 26, 19–30 (2013). 23867226 10.1016/j.devcel.2013.06.012 PMC3714589

[16] S. Bowling, A. D. Gregorio, M. Sancho, S. Pozzi, M. Aarts, M. Signore, M. D. Schneider, J. P. Martinez-Barbera, J. Gil, T. A. Rodríguez, P53 and mTOR signalling determine fitness selection through cell competition during early mouse embryonic development. Nat. Commun. 9, 1763 (2018). 29720666 10.1038/s41467-018-04167-y PMC5932021

[17] A. Lima, G. Lubatti, J. Burgstaller, D. Hu, A. P. Green, A. Di Gregorio, T. Zawadzki, B. Pernaute, E. Mahammadov, S. Perez-Montero, M. Dore, J. M. Sanchez, S. Bowling, M. Sancho, T. Kolbe, M. M. Karimi, D. Carling, N. Jones, S. Srinivas, A. Scialdone, T. A. Rodriguez, Cell competition acts as a purifying selection to eliminate cells with mitochondrial defects during early mouse development. Nat. Metab. 3, 1091–1108 (2021). 34253906 10.1038/s42255-021-00422-7 PMC7611553

[18] A. Suomalainen, J. Nunnari, Mitochondria at the crossroads of health and disease. Cell 187, 2601–2627 (2024). 38788685 10.1016/j.cell.2024.04.037

[19] A. Lima, J. Burgstaller, J. M. Sanchez-Nieto, T. A. Rodríguez, The mitochondria and the regulation of cell fitness during early mammalian development. Curr. Top. Dev. Biol. 128, 339–363 (2018). 29477168 10.1016/bs.ctdb.2017.10.012

[20] K. Pakos-Zebrucka, I. Koryga, K. Mnich, M. Ljujic, A. Samali, A. M. Gorman, The integrated stress response. EMBO Rep. 17, 1374–1395 (2016). 27629041 10.15252/embr.201642195 PMC5048378

[21] M. Costa-Mattioli, P. Walter, The integrated stress response: From mechanism to disease. Science 368, eaat5314 (2020). 32327570 10.1126/science.aat5314 PMC8997189

[22] M. Amiri, S. J. Kiniry, A. P. Possemato, N. Mahmood, T. Basiri, C. R. Dufour, N. Tabatabaei, Q. Deng, M. A. Bellucci, K. Harwalkar, M. P. Stokes, V. Giguere, R. J. Kaufman, Y. Yamanaka, P. V. Baranov, S. Tahmasebi, N. Sonenberg, Impact of eIF2α phosphorylation on the translational landscape of mouse embryonic stem cells. Cell Rep. 43, 113615 (2024). 38159280 10.1016/j.celrep.2023.113615 PMC10962698

[23] B. Pernaute, S. Pérez-Montero, J. M. Sánchez Nieto, A. Di Gregorio, A. Lima, K. Lawlor, S. Bowling, G. Liccardi, A. Tomás, P. Meier, H. Sesaki, G. A. Rutter, I. Barbaric, T. A. Rodríguez, DRP1 levels determine the apoptotic threshold during embryonic differentiation through a mitophagy-dependent mechanism. Dev. Cell 57, 1316–1330.e7 (2022). 35597240 10.1016/j.devcel.2022.04.020 PMC9297746

[24] K. Labbé, L. LeBon, B. King, N. Vu, E. H. Stoops, N. Ly, A. Lefebvre, P. Seitzer, S. Krishnan, J.-M. Heo, B. Bennett, C. Sidrauski, Specific activation of the integrated stress response uncovers regulation of central carbon metabolism and lipid droplet biogenesis. Nat. Commun. 15, 8301 (2024). 39333061 10.1038/s41467-024-52538-5 PMC11436933

[25] T. L. Keller, D. Zocco, M. S. Sundrud, M. Hendrick, M. Edenius, J. Yum, Y. J. Kim, H. K. Lee, J. F. Cortese, D. F. Wirth, J. D. Dignam, A. Rao, C. Y. Yeo, R. Mazitschek, M. Whitman, Halofuginone and other febrifugine derivatives inhibit prolyl-tRNA synthetase. Nat. Chem. Biol. 8, 311–317 (2012). 22327401 10.1038/nchembio.790 PMC3281520

[26] C. D’Aniello, A. Fico, L. Casalino, O. Guardiola, G. Di Napoli, F. Cermola, D. De Cesare, R. Tatè, G. Cobellis, E. J. Patriarca, G. Minchiotti, A novel autoregulatory loop between the Gcn2-Atf4 pathway and l-proline metabolism controls stem cell identity. Cell Death Differ. 22, 1094–1105 (2015). 25857264 10.1038/cdd.2015.24 PMC4572871

[27] C. Díaz-Díaz, M. Torres, Insights into the quantitative and dynamic aspects of cell competition. Curr. Opin. Cell Biol. 60, 68–74 (2019). 31108429 10.1016/j.ceb.2019.04.003

[28] L. Ballesteros-Arias, V. Saavedra, G. Morata, Cell competition may function either as tumour-suppressing or as tumour-stimulating factor in *Drosophila*. Oncogene 33, 4377–4384 (2014). 24096487 10.1038/onc.2013.407

[29] P. K. Todorova, B. T. Jackson, V. Garg, K. I. Paras, J. S. Brunner, A. E. Bridgeman, Y. Chen, S. C. Baksh, J. Yan, A. K. Hadjantonakis, L. W. S. Finley, Amino acid intake strategies define pluripotent cell states. Nat. Metab. 6, 127–140 (2024). 38172382 10.1038/s42255-023-00940-6 PMC10842923

[30] L. Casalino, S. Comes, G. Lambazzi, B. De Stefano, S. Filosa, S. De Falco, D. De Cesare, G. Minchiotti, E. J. Patriarca, Control of embryonic stem cell metastability by l-proline catabolism. J. Mol. Cell Biol. 3, 108–122 (2011). 21307025 10.1093/jmcb/mjr001

[31] S. Comes, M. Gagliardi, N. Laprano, A. Fico, A. Cimmino, A. Palamidessi, D. De Cesare, S. De Falco, C. Angelini, G. Scita, E. J. Patriarca, M. R. Matarazzo, G. Minchiotti, L-proline induces a mesenchymal-like invasive program in embryonic stem cells by remodeling H3K9 and H3K36 methylation. Stem Cell Reports 1, 307–321 (2013). 24319666 10.1016/j.stemcr.2013.09.001 PMC3849245

[32] H. J. Glover, H. Holliday, R. A. Shparberg, D. Winkler, M. Day, M. B. Morris, Signalling pathway crosstalk stimulated by L-proline drives mouse embryonic stem cells to primitive-ectoderm-like cells. Development 150, dev201704 (2023). 37823343 10.1242/dev.201704 PMC10652046

[33] J. M. Washington, J. Rathjen, F. Felquer, A. Lonic, M. D. Bettess, N. Hamra, L. Semendric, B. S. Tan, J. A. Lake, R. A. Keough, M. B. Morris, P. D. Rathjen, L-proline induces differentiation of ES cells: A novel role for an amino acid in the regulation of pluripotent cells in culture. Am. J. Physiol. Cell Physiol. 298, C982–C992 (2010). 20164384 10.1152/ajpcell.00498.2009

[34] C. Rhiner, J. M. López-Gay, D. Soldini, S. Casas-Tinto, F. A. Martín, L. Lombardía, E. Moreno, Flower forms an extracellular code that reveals the fitness of a cell to its neighbors in *Drosophila*. Dev. Cell 18, 985–998 (2010). 20627080 10.1016/j.devcel.2010.05.010

[35] E. Madan, C. J. Pelham, M. Nagane, T. M. Parker, R. Canas-Marques, K. Fazio, K. Shaik, Y. Yuan, V. Henriques, A. Galzerano, T. Yamashita, M. A. F. Pinto, A. M. Palma, D. Camacho, A. Vieira, D. Soldini, H. Nakshatri, S. R. Post, C. Rhiner, H. Yamashita, D. Accardi, L. A. Hansen, C. Carvalho, A. L. Beltran, P. Kuppusamy, R. Gogna, E. Moreno, Flower isoforms promote competitive growth in cancer. Nature 572, 260–264 (2019). 31341286 10.1038/s41586-019-1429-3

[36] S. N. Meyer, M. Amoyel, C. Bergantinos, C. de la Cova, C. Schertel, K. Basler, L. A. Johnston, An ancient defense system eliminates unfit cells from developing tissues during cell competition. Science 346, 1258236 (2014). 25477468 10.1126/science.1258236 PMC5095928

[37] C. C. Soares, A. Rizzo, M. F. Maresma, P. Meier, Autocrine glutamate signaling drives cell competition in *Drosophila*. Dev. Cell 59, 2974–2989.e5 (2024). 39047739 10.1016/j.devcel.2024.06.022

[38] A. R. Banreti, P. Meier, The NMDA receptor regulates competition of epithelial cells in the *Drosophila* wing. Nat. Commun. 11, 2228 (2020). 32376880 10.1038/s41467-020-16070-6 PMC7203100

[39] P. Simpson, Parameters of cell competition in the compartments of the wing disk of *Drosophila*. Dev. Biol. 69, 182–193 (1979). 446891 10.1016/0012-1606(79)90284-7

[40] J. Schindelin, I. Arganda-Carreras, E. Frise, V. Kaynig, M. Longair, T. Pietzsch, S. Preibisch, C. Rueden, S. Saalfeld, B. Schmid, J. Y. Tinevez, D. J. White, V. Hartenstein, K. Eliceiri, P. Tomancak, A. Cardona, Fiji: An open-source platform for biological-image analysis. Nat. Methods 9, 676–682 (2012). 22743772 10.1038/nmeth.2019 PMC3855844

[41] D. Kim, G. Pertea, C. Trapnell, H. Pimentel, R. Kelley, S. L. Salzberg, TopHat2: Accurate alignment of transcriptomes in the presence of insertions, deletions and gene fusions. Genome Biol. 14, R36 (2013). 23618408 10.1186/gb-2013-14-4-r36 PMC4053844

[42] M. I. Love, W. Huber, S. Anders, Moderated estimation of fold change and dispersion for RNA-seq data with DESeq2. Genome Biol. 15, 550 (2014). 25516281 10.1186/s13059-014-0550-8 PMC4302049

[43] J. I. MacRae, M. W. Dixon, M. K. Dearnley, H. H. Chua, J. M. Chambers, S. Kenny, I. Bottova, L. Tilley, M. J. McConville, Mitochondrial metabolism of sexual and asexual blood stages of the malaria parasite *Plasmodium falciparum*. BMC Biol. 11, 67 (2013). 23763941 10.1186/1741-7007-11-67 PMC3704724

