# Supplementary material for: Sensing of extracellular l-proline availability by the integrated stress response determines the outcome of cell competition
Source: Sci Adv. 2025 Jul 9;11(28):eadw1883. doi: 10.1126/sciadv.adw1883 (PMC12239963; doi:10.1126/sciadv.adw1883)
Supplement: Supplementary file 1 — Figs. S1 to S10 Tables S1 and S2 Legend for table S3 [file sciadv.adw1883_sm.pdf]

Science Advances
AAAS

Supplementary Materials for

Sensing of extracellular L-proline availability by the integrated stress response determines the outcome of cell competition

Shruthi Krishnan *et al.*

Corresponding author: Tristan A. Rodriguez, tristan.rodriguez@imperial.ac.uk

Sci. Adv. **11**, eadw1883 (2025)
DOI: 10.1126/sciadv.adw1883

The PDF file includes:

Figs. S1 to S10
Tables S1 and S2
Legend for table S3

Other Supplementary Material for this manuscript includes the following:

Table S3

Supplementary Figure 1

A

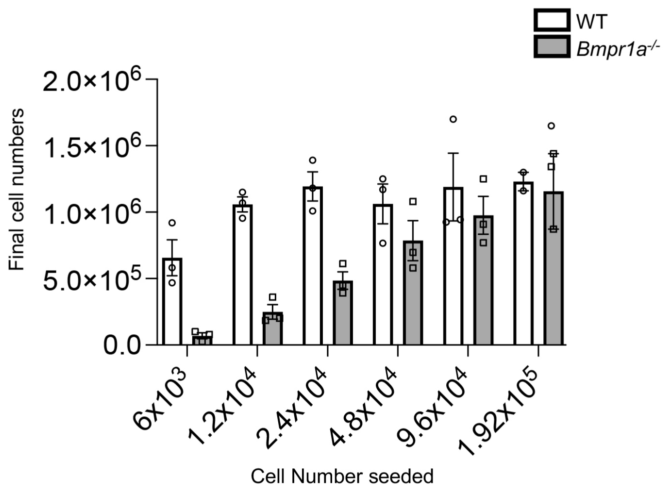

Supplementary Figure 1. Bar graph representing final cell numbers of wild-type (WT) and *Bmpr1a*$^{-/-}$ cells when seeded at different cell densities. n=3. Error bars denote SEM. **$p < 0.01$, *$p < 0.05$, unpaired t-test.

Supplementary Figure 2

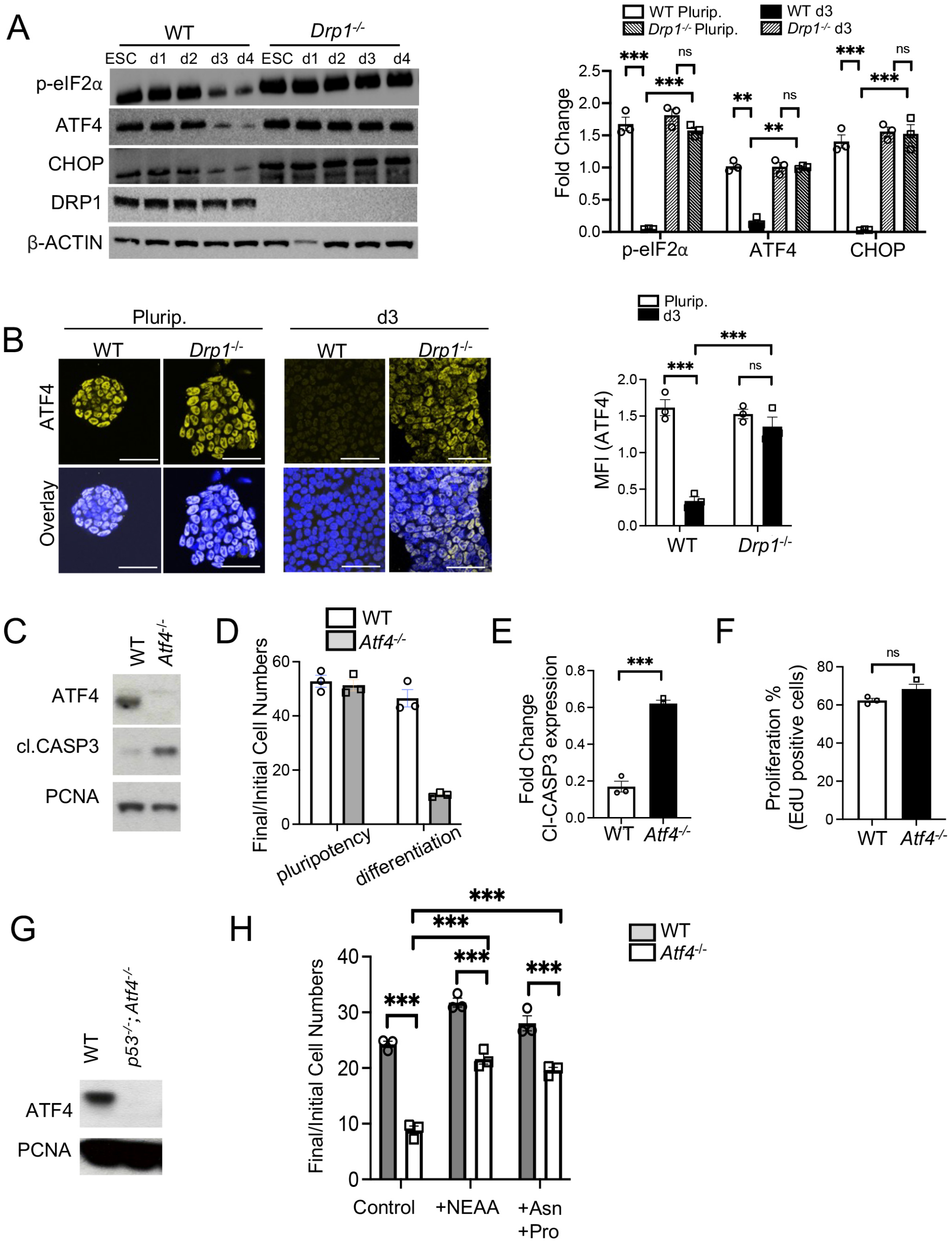

Supplementary Figure 2. (A) Wildtype (WT) and *Drp1*$^{-/-}$ cells were analysed by immunoblot after culture in pluripotency conditions (ESC) or at different time-points of differentiation (d1-d4) to detect expression levels of p-eIF2a, ATF4, CHOP and b-ACTIN (loading control). Bar graph represents fold change in expression levels of p-eIF2a, ATF4 and CHOP in ESC and d3 cells. (B) Immunofluorescence analysis of ATF4 (yellow) in ESC and d3 WT and *Drp1*$^{-/-}$ cells, nuclei are counterstained with Hoechst (blue). Scale bars= 100uM. Bar graph represents quantification of staining intensity of ATF4 (MFI in arbitrary units) in the cells. (C) immunoblot analysis showing ATF4 and cleaved-CASPASE 3 expression in WT and *Atf4*$^{-/-}$ cells. PCNA is used as a loading control. (D) Bar graph depicting ratio of final (day 4) to initial cell numbers of WT and *Atf4*$^{-/-}$ cells cultured in pluripotency and differentiation conditions. € Quantification of cleaved-CASPASE 3 levels from (C). (F) Proliferation was assessed by EdU incorporation assay and bar graph depicts % of EdU incorporation in WT and *Atf4*$^{-/-}$ cells. (G) immunoblot analysis showing ATF4 expression in WT and *p53*$^{-/-}$; *Atf4*$^{-/-}$ cells. PCNA was used as a loading control. (H) Bar graph representing ratio of final (d3) to initial cell numbers of WT and *Atf4*$^{-/-}$ cells cultured in control medium and in the presence of excess of non-essential amino acids (+NEAA) and excess of Asparagine and Proline (+Asn+Pro). n=3 for all studies. Error bars denote SEM. *** $p < 0.005$, **$p < 0.01$, *$p < 0.05$, ns-non-significant; two-way ANOVA and Tukey's post-hoc test (A, B, H, F). *** $p < 0.005$, ns-non-significant; unpaired t-test (D, E).

Supplementary Figure 3

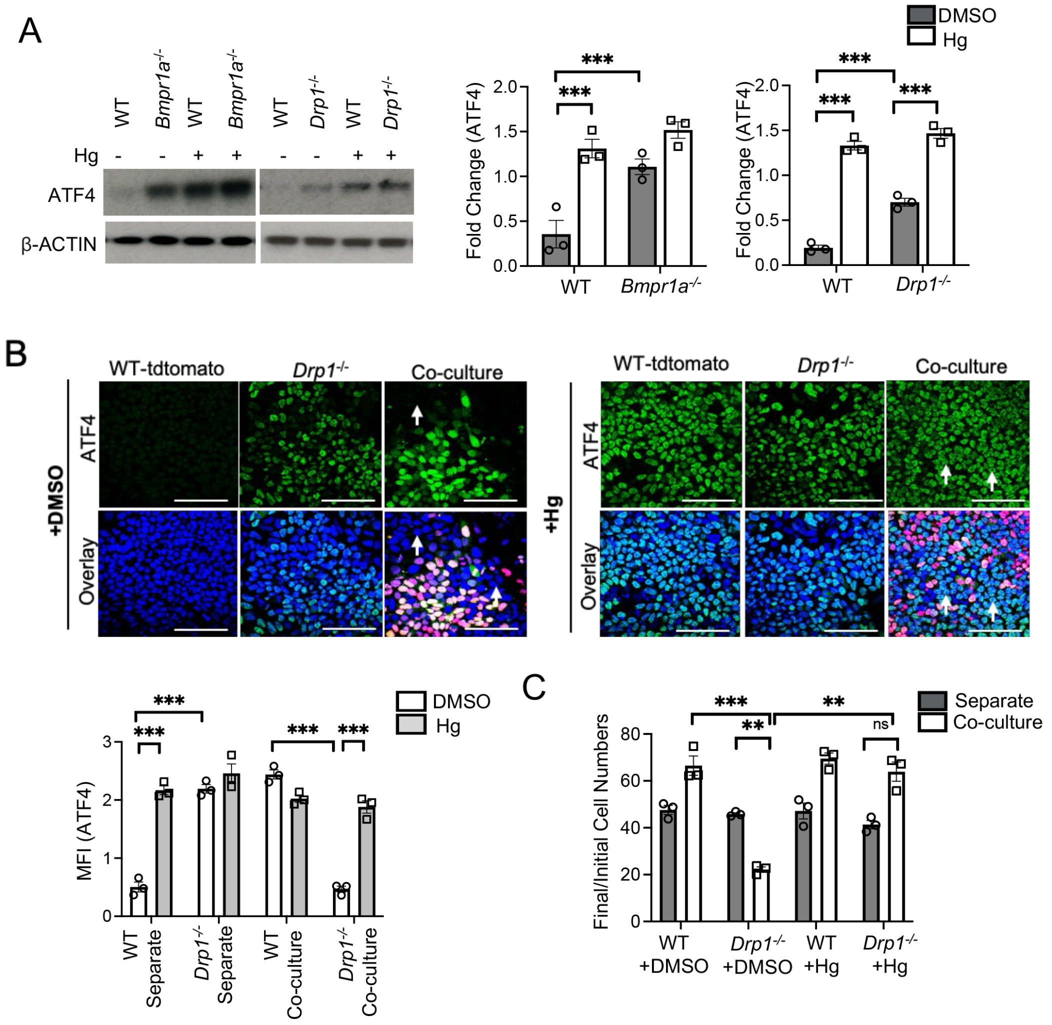

Supplementary Figure 3. (A) Wild-type (WT), *Bmpr1a*$^{-/-}$ and *Drp1*$^{-/-}$ cells were cultured in differentiation conditions and treated with DMSO or halofuginone (+Hg; 20nM) for 48h. Immunoblot analysis was performed to analyse expression levels of ATF4, b-ACTIN (loading control). Bar graphs represent densitometry-based quantification of ATF4 expression as fold change. (B) Immunofluorescence analysis of ATF4 (green) in WT-tdtomato (red), *Drp1*$^{-/-}$ cells in separate and co-cultures after treatment with DMSO or Hg for 48h (d1-d3). Nuclei are counterstained with Hoechst (blue), white arrows indicate ATF4 expression levels in *Drp1*$^{-/-}$ cells in co-cultures. Scale bars= 100uM. Bar graph represents quantification of staining intensity of ATF4 (MFI in arbitrary units) in the cells. (C) Cell competition assays between WT and *Drp1*$^{-/-}$ cells cultured for 48h with DMSO or Hg. The bar graph depicts the ratio of final cell numbers (day 3) to initial cell numbers for separate and co-cultures of these cells. n=3 for all studies. Error bars denote SEM. *** $p < 0.005$, **$p < 0.01$ ns-non-significant; two-way ANOVA and Tukey's post-hoc test.

Supplementary Figure 4

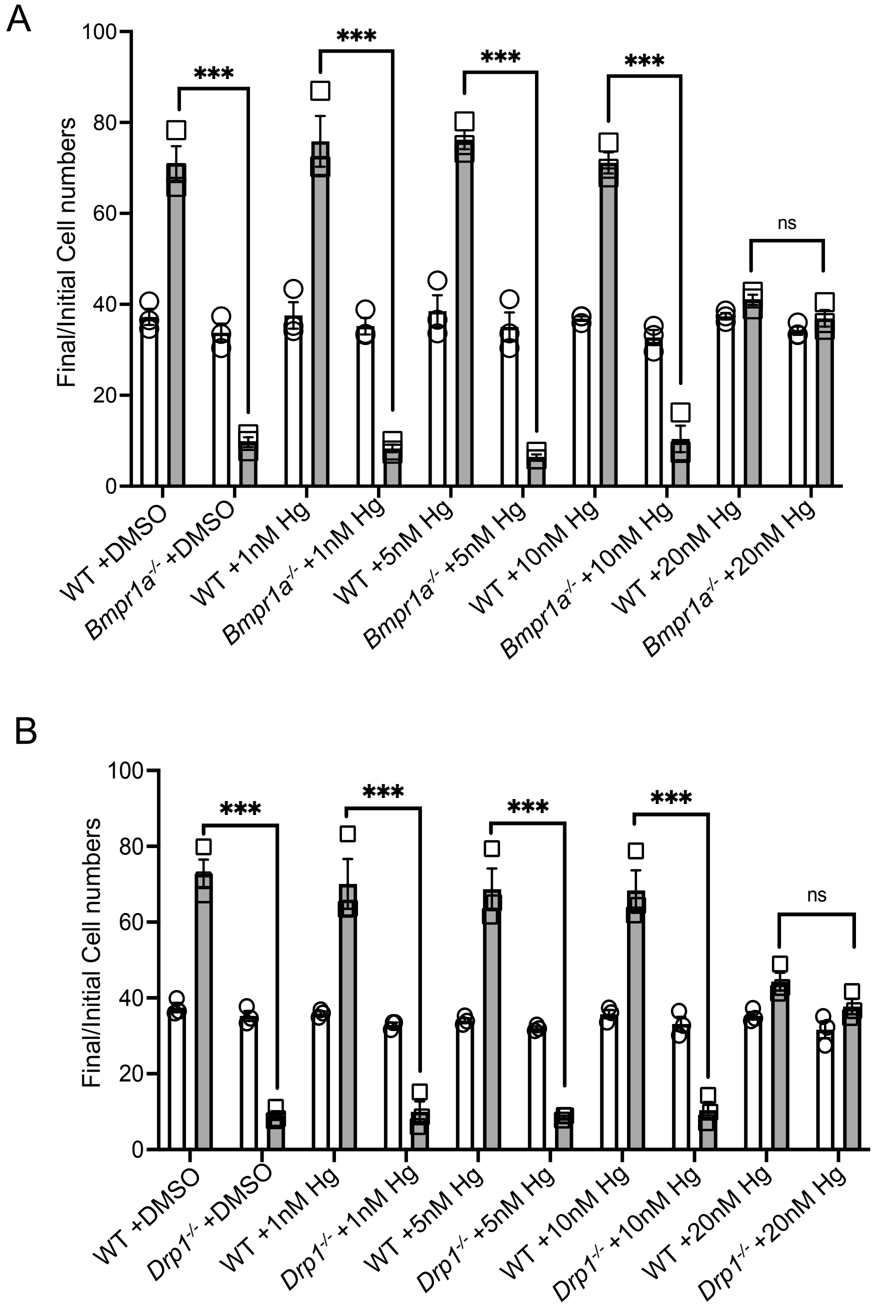

Supplementary Figure 4. Cell competition assays between (A) WT, *Bmpr1a*$^{-/-}$ and (B) WT, *Drp1*$^{-/-}$ cells cultured for 48h in DMSO or Hg treated media depicted as the ratio of final (day 3) cell numbers to the initial cell numbers of the cells in separate and co-cultures. n=3 for all studies. Error bars denote SEM. *** $p < 0.005$, ns-non-significant; two-way ANOVA and Tukey's post-hoc test.

Supplementary Figure 5

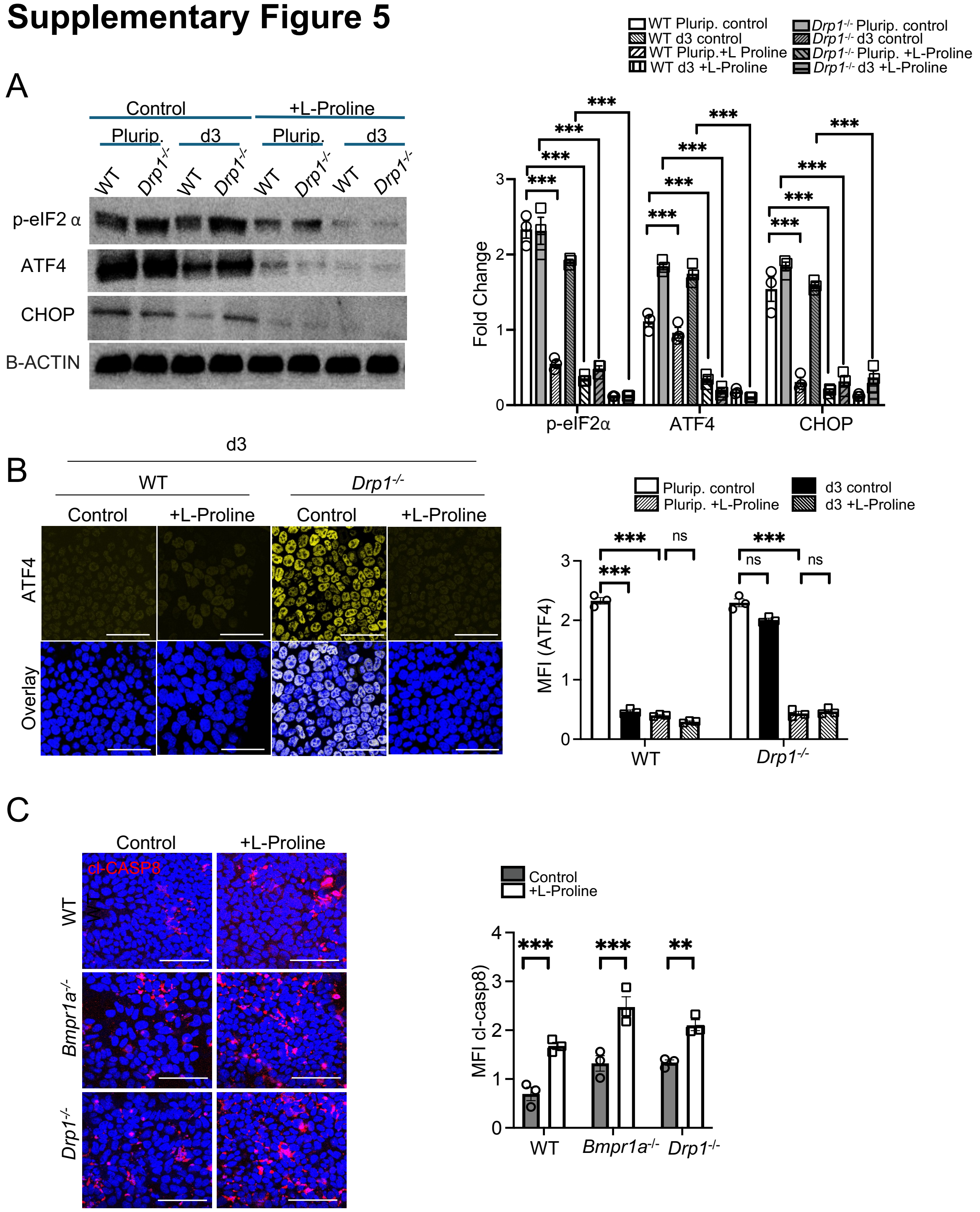

Supplementary Figure 5. (A) Wild-type (WT) and *Drp1*$^{-/-}$ cells were cultured in pluripotency (ESC) or differentiations (d3) conditions in control media or media supplemented with L-Proline for 48h (0.5mM; +L-Proline). Expression of ISR proteins, p-eIF2a, ATF4, CHOP was analysed by immunoblots. b-ACTIN was used as a loading control. Bar graph represents fold change in expression of the proteins in the various conditions. (B) Immunofluorescence analysis of ATF4 in WT and *Drp1*$^{-/-}$ at day 3 of differentiation cultured in control medium or medium supplemented with L-Proline for 48h. Scale bar=100uM. Bar graph represents quantification of staining intensity of ATF4. (C) Immunofluorescence analysis of cleaved caspase 8 (cl. CASP8) in WT *Bmpr1a*$^{-/-}$ and *Drp1*$^{-/-}$ cells cultured in control medium or medium supplemented with L-Proline for 48h. Scale bar=100uM. Bar graph represents quantification of staining intensity of cl. CASP8 (MFI in arbitrary units) in the cells. n=3 for all studies. Error bars denote SEM. *** $p < 0.005$, *$p < 0.05$ two-way ANOVA and Tukey's post-hoc test.

Supplementary Figure 6

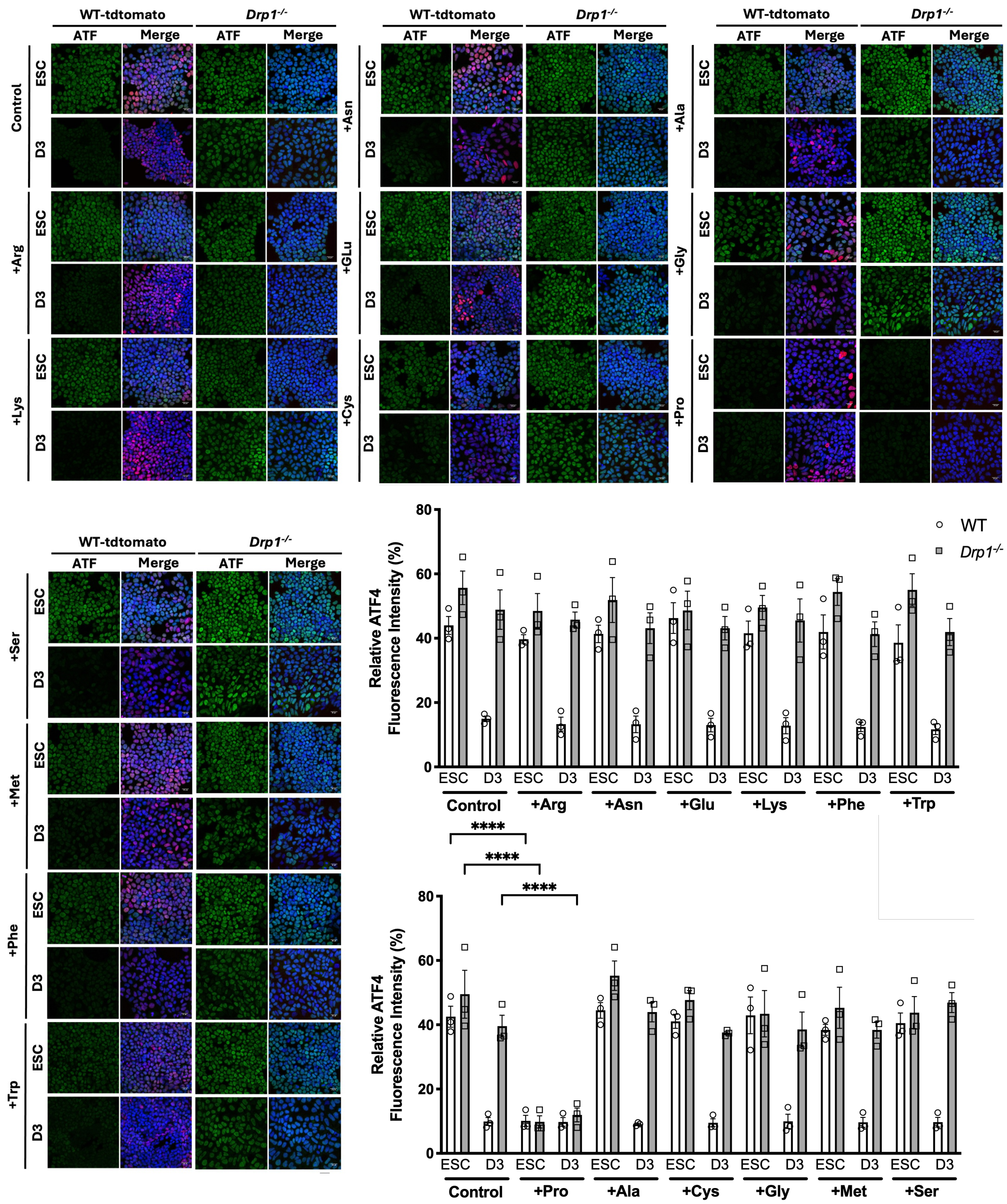

Supplementary Figure 6. (A) Wild-type (WT) and *Drp1*$^{-/-}$ cells were cultured in pluripotency (ESC) or differentiations (d3) conditions in control media or media supplemented with different amino acids; arginine (+Arg), lysine (+Lys), asparagine (+Asn), glutamate (+Glu), cysteine (+Cys), alanine (+Ala), glycine (+Gly), Proline (+Pro), Serine (+Ser), Methionine (+Met), Phenylalanine (+Phe) and Tryptophan (+Try) for 48h (all amino acids-0.5mM). Immunofluorescence analysis of ATF4 (green) in ESC and d3 WT and *Drp1*$^{-/-}$ cells, nuclei are counterstained with Hoechst (blue). Scale bars= 20um. (B) Bar graph represents quantification of relative staining intensity of ATF4 (in %). n=3 for all studies. Error bars denote SEM. ****p<0.005 two-way ANOVA and Tukey's post hoc test.

Supplementary Figure 7

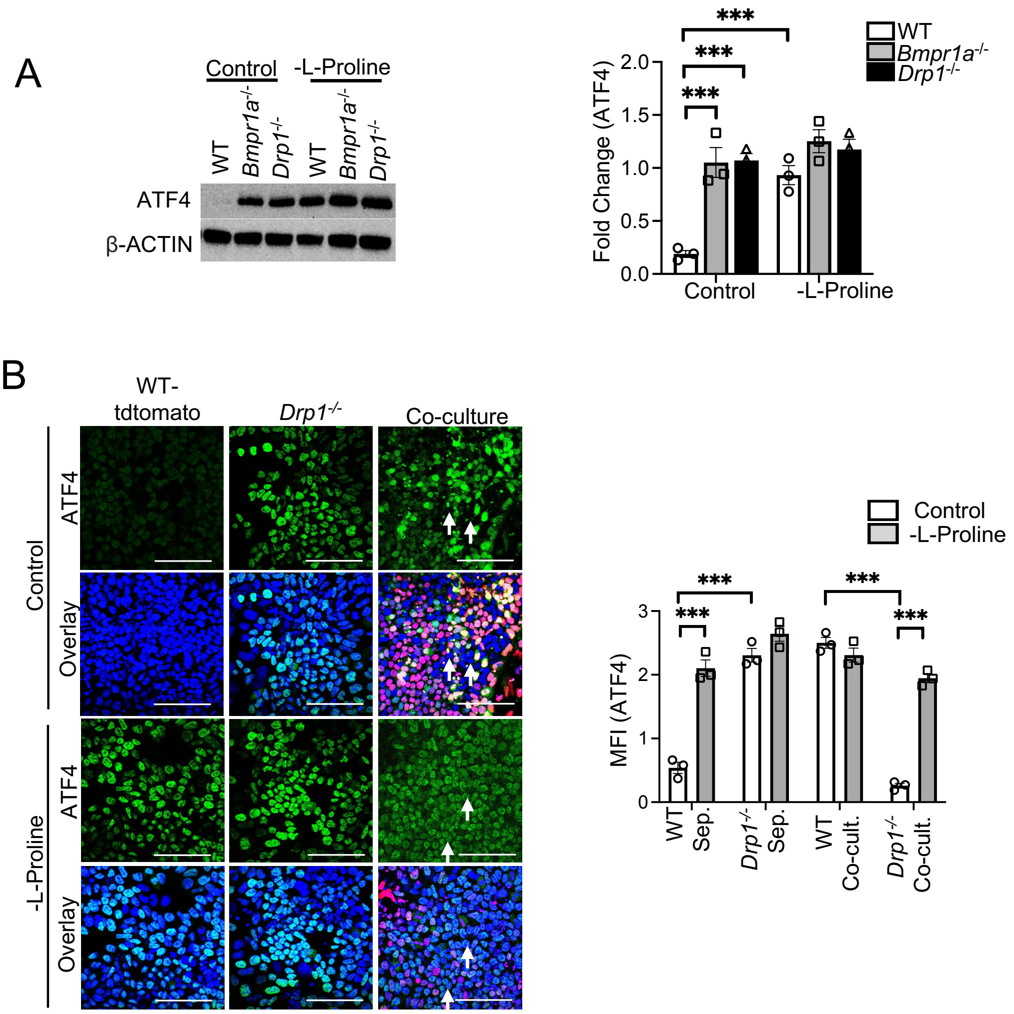

Supplementary Figure 7. (A) Wild-type (WT), *Bmpr1a*$^{-/-}$ and *Drp1*$^{-/-}$ cells were cultured in control differentiation medium and medium deprived of L-Proline (-L-Proline). Immunoblot analysis was performed to analyse expression levels of ATF4 and b-ACTIN (loading control) in these cells. Bar graphs represent densitometry-based quantification of ATF4 expression as a fold change.

(B) Immunofluorescence analysis of ATF4 (green) in WT-tdtomato (red), *Drp1*$^{-/-}$ cells in separate and co-cultures in control and -L-Proline medium. Nuclei are counterstained with Hoechst (blue), white arrows indicate ATF4 expression levels in *Drp1*$^{-/-}$ cells in co-cultures. Scale bars= 100uM. Bar graph represents quantification of staining intensity of ATF4 (MFI in arbitrary units) in the cells. n=3 for all studies. Error bars denote SEM. *** $p < 0.005$, *$p < 0.05$ two-way ANOVA and Tukey's post-hoc test.

Supplementary Figure 8

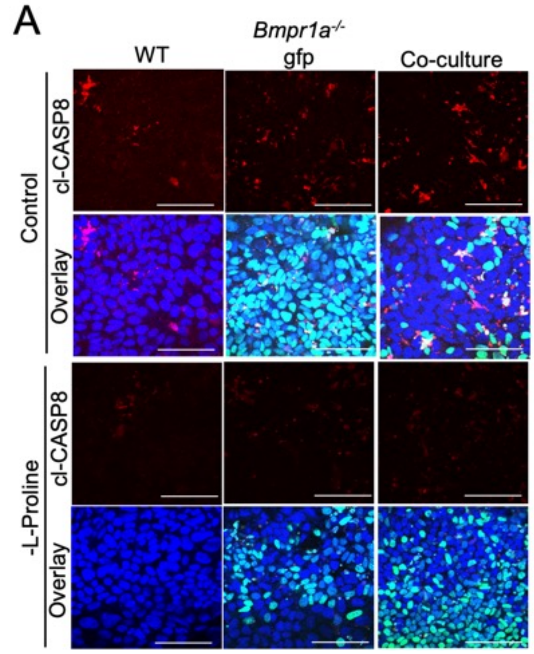

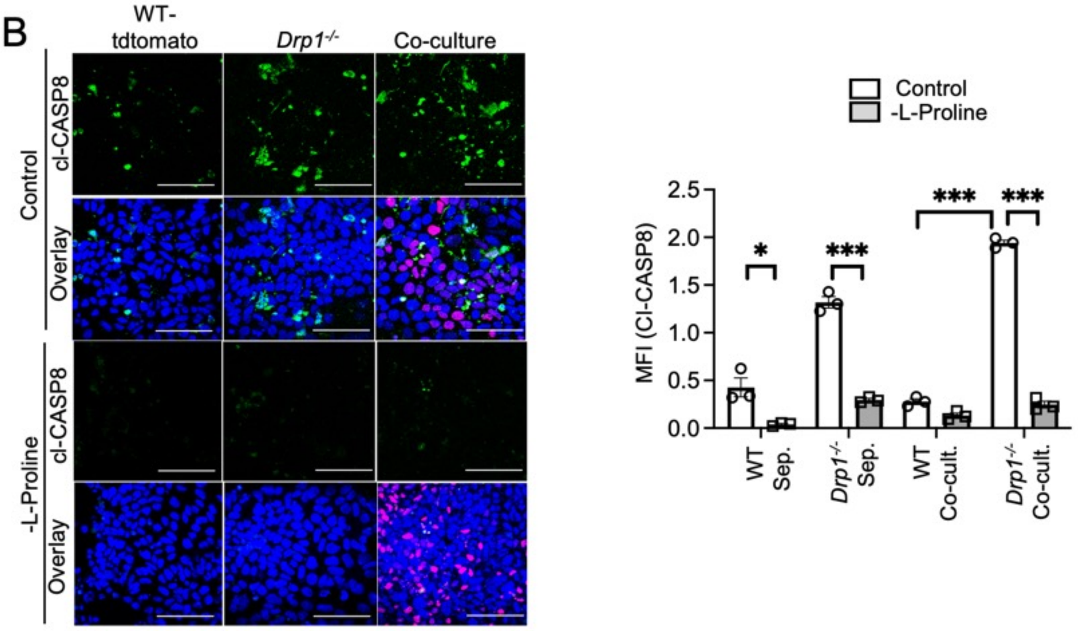

Supplementary Figure 8. (A) Immunofluorescence analysis of cleaved-CASPASE8 (red) in WT and *Bmpr1a*$^{-/-}$ (green) cells in separate and co-cultures in control and -L-Proline medium. Nuclei are counterstained with Hoechst (blue). Scale bars= 100uM. (B) Immunofluorescence analysis of cleaved-CASPASE8 (green) in WT tdtomato (red) and *Drp1*$^{-/-}$ cells in separate and co-cultures in control and -L-Proline medium. Nuclei are counterstained with Hoechst (blue). Scale bars= 100uM. Bar graph represents quantification of staining intensity of cleaved CASPASE8 (MFI in arbitrary units) in the cells. n=3. Error bars denote SEM. *** $p < 0.005$, *$p < 0.05$ two-way ANOVA and Tukey's post-hoc test.

Supplementary Figure 9

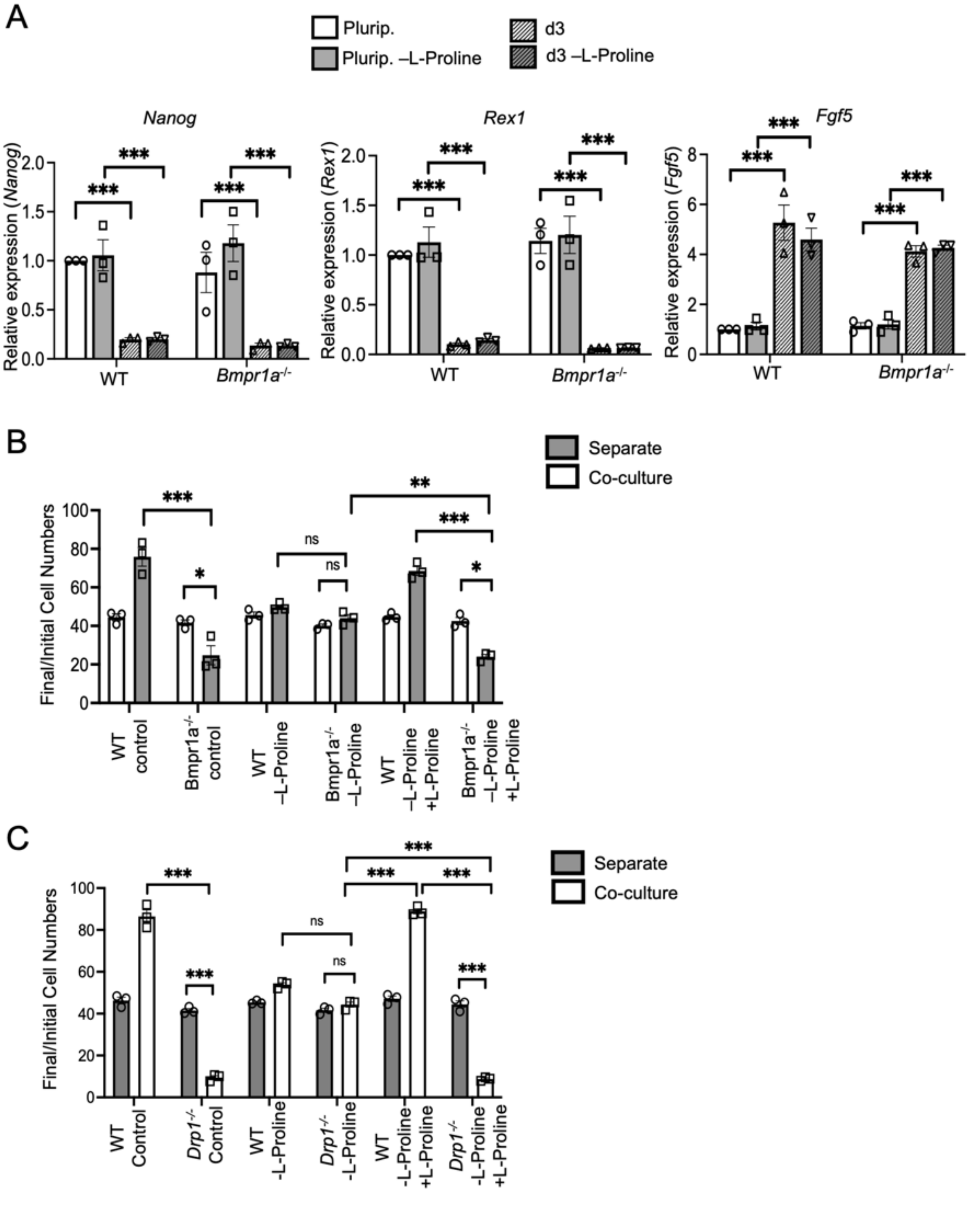

Supplementary Figure 9. (A) Quantitative RT-PCR showing gene expression levels of pluripotency (*Nanog* and *Rex1*) and differentiation markers (*Fgf5*) in wild-type (WT) and *Bmpr1a*$^{-/-}$ ESCs in pluripotency and at day 3 of differentiation. Gene expression is normalized against *Gapdh*. (B) Cell competition assays between WT and *Bmpr1a*$^{-/-}$ cells in media lacking L-Proline with or without L-Proline treatment (0.25mM, 48h d1-d3), depicted as ratio of final cell numbers (day 3) to initial cell numbers for separate and co-cultures of these cells. (C) Cell competition assays between WT and *Drp1*$^{-/-}$ cells lacking L-Proline with or without L-Proline treatment (0.25mM, 48h d1-d3), depicted as ratio of final cell numbers (day 3) to initial cell numbers for separate and co-cultures of these cells. n=3 for all studies. Error bars denote SEM. *** $p < 0.005$, *$p < 0.05$ two-way ANOVA and Tukey's post-hoc test.

Supplementary Figure 10

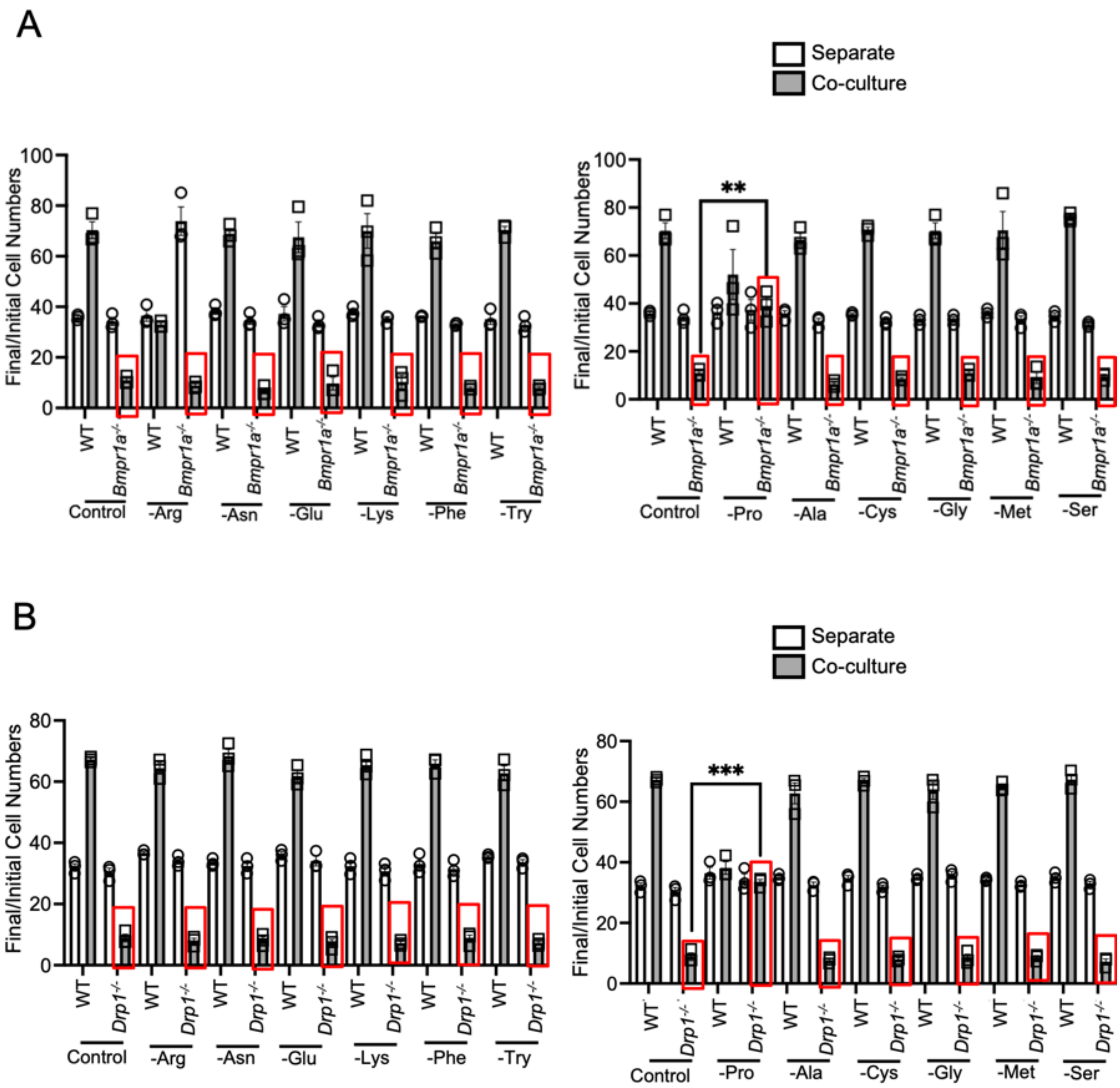

Supplementary Figure 10. (A) Cell competition assays between WT and *Bmpr1a*$^{-/-}$ cells in control medium and media lacking different amino acids, arginine (-Arg), asparagine (-Asn), glutamate (-Glu), lysine (-Lys), phenylalanine (-Phe), tryptophan (-Try), proline (-Pro), alanine (-Ala), cysteine (-Cys), glycine (-Gly), methionine (-Met), serine (-Ser), depicted as ratio of final cell numbers (day 3) to initial cell numbers (day 0) for separate and co-cultures of these cells. (B) Cell competition assays between WT and *Drp1*$^{-/-}$ cells in control medium and media lacking different amino acids, arginine (-Arg), asparagine (-Asn), glutamate (-Glu), lysine (-Lys), phenylalanine (-Phe), tryptophan (-Try), proline (-Pro), alanine (-Ala), cysteine (-Cys), glycine (-Gly), methionine (-Met), serine (-Ser), depicted as ratio of final cell numbers (day 3) to initial cell numbers (day 0) for separate and co-cultures of these cells. n=3 for all studies. Error bars denote SEM. *** $p < 0.005$, **$p < 0.01$, two-way ANOVA and Tukey's post-hoc test.

Supplementary Table 1

Amino Acids	Final Concentration (mM)	Amino Acids	Final Concentration (mM)
Glycine	0.65	L-Lysine hydrochloride	1.30
L-Alanine	0.07	L-Methionine	0.32
L-Arginine hydrochloride	1.10	L-Phenylalanine	0.62
L-Asparagine-H2O	0.06	L-Serine	0.65
L-Aspartic acid	0.05	L-Threonine	1.25
L-Cysteine hydrochloride-H2O	0.10	L-Tryptophan	0.12
L-Cystine 2HCl	0.10	L-Tyrosine disodium salt dihydrate	0.61
L-Glutamic Acid	0.05	L-Valine	0.80
L-Glutamine	2.50	L-Lysine hydrochloride	1.30
L-Histidine hydrochloride-H2O	0.35		

Supplementary Table 1: Composition of -L-Proline medium.

Supplementary Table 2

Primer (gene)	Forward primer (5'-3')	Reverse primer (5'-3')
Nanog	CTTACAAGGGTCTGCTACTGAGATGC	TGCTTCCTGGCAAGGACCTT
Rex1	CGAGTGGCAGTTTCTTCTTGG	GACTCACTTCCAGGGGGCAC
Fgf5	AAAGTCAATGGCTCCCACGAA	CTTCAGTCTGTACTTCACTGG
Slc38a2	CCACCTCTTCTGCCTGTTGT	GCTTCCTTTTGTCCTTGGCG
Aldh18a1	CGAGCAGAAGCGCAGAAATC	CCTTAACACTAATCACCCCCTG A
Pycr1	CCAGCTCAAGGACAACGTCT	ACTGCAGTTCTCTGGTTCGG
Gapdh	CATGGCCTTCCGTGTTCCTA	GCGGCACGTCAGATCCA

Supplementary Table 2: List of primers used for qPCR analysis.

Supplementary Table 3 (excel file)

List of differentially expressed genes between *Atf4*$^{-/-}$, WT cells and *p53*$^{-/-}$ *Atf4*$^{-/-}$, *p53*$^{-/-}$ cells.
